# Coupled Effects of Reprocessing, TiO_2_, and Fused Filament Fabrication on Polylactic Acid Weathering Durability: A Critical Review

**DOI:** 10.3390/polym18212795

**Published:** 2026-11-08

**Authors:** Thomas Rodríguez-Peña, Edwin Collado, Yessica Saez, Nacari Marin-Calvo

**Affiliations:** 1 Universidad Tecnológica de Panamá, Panama City 0819-07289, Panama; thomas.rodriguez@utp.ac.pa (T.R.-P.); yessica.saez@utp.ac.pa (Y.S.); 2 Departamento de Ciencia de los Materiales, Facultad de Ingeniería Mecánica, Centro Regional de Azuero, Universidad Tecnológica de Panamá, Panama City 0819-07289, Panama; 3 Departamento de Ingeniería Electrónica y Telecomunicaciones, Facultad de Ingeniería Eléctrica, Centro Regional de Azuero, Universidad Tecnológica de Panamá, Panama City 0819-07289, Panama; 4 Grupo de Investigación en Ingeniería, Tecnología y Sistemas Inteligentes Aplicados a la Sociedad (ITSIAS), Centro Regional de Azuero, Universidad Tecnológica de Panamá, Panama City 0819-07289, Panama; 5 Centro de Estudios Multidisciplinarios en Ciencias, Ingeniería y Tecnología (CEMCIT AIP), Panama City 0819-07289, Panama

**Keywords:** fused filament fabrication, hydrolysis, polylactic acid, recycled PLA, titanium dioxide, tropical climate, ultraviolet degradation, weathering durability

## Abstract

Titanium dioxide (TiO_2_) is incorporated into polylactic acid (PLA) to improve stiffness, promote crystallization, and provide ultraviolet (UV) shielding. However, its influence on durability is complex, as photocatalytic activity may accelerate degradation under environmental exposure. This review examines how TiO_2_ characteristics, melt processing history, fused filament fabrication (FFF) architecture, and exposure conditions collectively influence PLA durability. Primary studies and previous reviews are critically compared based on material formulation, processing conditions, specimen geometry, environmental exposure, and measured properties. Evidence indicates that reprocessing generally reduces molar mass and melt viscosity, while crystallization and printing conditions may partially preserve mechanical performance. TiO_2_ can enhance selected properties, although its effects depend on particle characteristics, dispersion, and surface accessibility. Furthermore, interlayer interfaces and connected porosity influence moisture transport and damage localization, limiting the applicability of findings from films and moulded specimens to FFF components. While existing reviews have addressed PLA–TiO_2_ composites and their properties, the combined influence of reprocessing, TiO_2_ incorporation, FFF architecture, and tropical weathering has received limited attention. Unlike previous reviews that primarily examine these factors separately, this review integrates their potential interactions to provide a processing-history-dependent perspective on PLA durability. It also identifies research priorities involving controlled processing comparisons, multiscale characterization, and complementary natural and accelerated weathering tests to support more reliable assessments of outdoor performance.

## 1. Introduction

Polylactic acid (PLA) is widely used in fused filament fabrication (FFF) because of its ease of processing, relatively low printing temperatures, and availability from renewable resources. These characteristics make it an attractive material for producing prototypes and functional components through additive manufacturing. However, its susceptibility to degradation under elevated temperatures, humidity, and solar radiation can limit its durability in outdoor applications. To address some of these limitations, titanium dioxide (TiO_2_) has been incorporated into PLA to improve properties such as stiffness, thermal stability, and UV protection. Nevertheless, these improvements do not necessarily guarantee greater long-term durability, as the performance of PLA–TiO_2_ composites also depends on processing conditions, particle characteristics, printed structure, and environmental exposure.

### 1.1. Factors Affecting PLA Durability

As an aliphatic polyester, PLA exhibits properties that depend on several factors, including stereochemistry, molar mass distribution, crystallinity, thermal history, additives, and processing conditions [1,2,3,4]. Its chemical structure makes it susceptible to hydrolytic degradation under suitable conditions, contributing to its degradation at the end of its useful life. However, this same susceptibility can reduce melt viscosity during processing and accelerate embrittlement when the material is exposed to warm and humid environments [5,6,7].

It is important to distinguish between the terms bio-based, biodegradable, and durable, as they describe different material characteristics. Bio-based refers to the partial or complete derivation of a material from renewable biological resources; biodegradable describes its ability to undergo microbial degradation under specified environmental conditions and timescales; and durable refers to its ability to maintain the structural and functional properties required throughout its intended period of use [4,5,7]. These terms should not be used interchangeably. Biodegradability under suitable end-of-life conditions does not necessarily imply poor durability during use, nor does a bio-based origin guarantee biodegradability under ambient environmental conditions.

The durability of PLA also depends on how it is manufactured. Material extrusion additive manufacturing produces three-dimensional components through the successive deposition of material dispensed through a nozzle or orifice [8]. In FFF, the polymer is heated, extruded, and deposited layer by layer, forming bead boundaries, interlayer interfaces, infill structures, and voids that differ from those found in injection-moulded or compression-moulded specimens [9,10,11,12,13]. These structural features can influence mechanical performance and environmental degradation, making it necessary to evaluate durability at both the material and printed-component levels [14,15,16].

Additional changes may occur when PLA undergoes repeated processing before printing. Mechanical reprocessing exposes the polymer to further thermal and mechanical stresses, which can reduce molar mass and melt viscosity through hydrolytic, thermal, and thermo-oxidative degradation, as well as shear-associated effects. The extent of these changes depends on moisture content, processing temperature, residence time, oxygen exposure, polymer grade, and stabilizers [6,17,18,19,20,21]. Reprocessing can also alter crystallization behavior. Although shorter polymer chains may crystallize more readily, increased crystallinity does not necessarily indicate improved material performance, as it may occur alongside reduced molecular entanglement, lower melt strength, and decreased ductility [19,20,22].

These changes are particularly relevant when additives are incorporated into reprocessed PLA. Their effects depend not only on their composition and concentration but also on the condition of the polymer before printing. This relationship is especially important for TiO_2_, which can modify the initial properties of PLA while also influencing its response to environmental exposure.

### 1.2. Effects of TiO_2_ in PLA

TiO_2_ is incorporated into PLA to modify properties such as stiffness, crystallization behavior, thermal response, and UV protection. It can also provide antimicrobial or photocatalytic functionality, depending on the formulation and intended application [23,24,25,26]. However, these effects depend on several factors, including particle size, crystalline phase, surface chemistry, concentration, dispersion, and processing conditions.

The crystalline phase of TiO_2_ is particularly important because anatase and rutile exhibit different optical and photocatalytic properties. Their electronic structures and surface reactivity influence their interactions with light and the surrounding material. Surface coatings and chemical modifications can also affect particle dispersion, adhesion to the polymer matrix, and the accessibility of photocatalytically active sites [24,27,28].

Before environmental exposure, well-dispersed TiO_2_ particles may improve stiffness, influence crystallization, and increase certain measures of thermal stability. Some studies also suggest that TiO_2_ can help compensate for mechanical property losses associated with PLA reprocessing, depending on particle concentration and dispersion [23,29,30,31]. However, improvements in these initial properties do not necessarily result in greater durability under outdoor conditions.

During exposure to UV radiation and moisture, photocatalytically active TiO_2_ surfaces may promote the formation of reactive species that contribute to polymer chain scission and surface degradation. These processes can lead to cracking, embrittlement, and changes in crystallinity, particularly when particles are poorly dispersed or their surfaces remain accessible to the surrounding environment [24,28,32].

The beneficial and potentially detrimental effects of TiO_2_ are not necessarily contradictory, as they may occur under different conditions or at different stages of environmental exposure. Differences in particle characteristics, initial polymer structure, processing conditions, exposure duration, and measured properties can help explain the different results reported in the literature [25,32]. Understanding these differences is important when evaluating the durability of PLA–TiO_2_ composites produced through FFF, particularly when the material is exposed to multiple environmental factors.

### 1.3. Weathering Stresses

The durability of PLA-based materials under outdoor conditions is influenced by several environmental factors that may act simultaneously. In this review, weathering refers to the physical, chemical, and structural changes that occur when materials are exposed to natural outdoor conditions or simulated environmental stresses, including solar radiation, oxygen, temperature, and moisture. Moisture exposure may involve humidity, condensation, rainfall, and repeated wet–dry cycles. Together, these factors can promote photolysis, photo-oxidation, hydrolysis, physical ageing, changes in crystallinity, and progressive material damage [33,34,35,36,37,38].

Weathering is evaluated here through changes in material structure and the retention of mechanical and functional properties. It should not be confused with biodegradation or industrial composting, which involve different conditions and degradation processes [5,7,36].

Accelerated laboratory tests are commonly used to evaluate weathering behavior under controlled conditions of UV radiation, temperature, and moisture. These tests allow materials to be compared under defined exposure conditions and can help identify degradation mechanisms. However, accelerated weathering does not provide a direct or universal relationship between laboratory exposure time and outdoor durability [39,40,41,42]. For example, ASTM G154 requires the reporting of exposure conditions, including lamp type, irradiance, temperature, and moisture cycles, because test results depend on the selected conditions [41].

Natural weathering introduces additional variability because environmental conditions differ across geographic locations and seasons. Solar radiation, temperature, humidity, rainfall, pollutants, and the duration of surface wetness can influence the rate and extent of material degradation [43]. Consequently, results obtained under one set of environmental conditions cannot necessarily be applied to other climates. This limitation is especially relevant in tropical regions, where high temperatures, intense solar radiation, elevated humidity, and frequent rainfall may expose materials to several degradation factors simultaneously. Previous studies have shown that UV radiation, temperature, and humidity influence the degradation of PLA films and FFF components, while the internal architecture of printed parts can also affect the retention of mechanical properties during exposure [33,44,45,46].

Despite these findings, comparisons between studies remain difficult because of differences in material formulations, specimen geometry, exposure protocols, and measurement intervals. Many investigations focus on a single environmental condition, use commercial filaments with incompletely reported formulations, or evaluate material properties at only a few exposure times [36,45,46]. In addition, few studies have examined the combined influence of controlled PLA reprocessing, well-characterized TiO_2_ particles, FFF architecture, and tropical weathering conditions, making it difficult to determine how these factors affect the long-term durability of printed components.

### 1.4. Review Aim and Scope

This review evaluates the coupled effects of PLA reprocessing, TiO_2_ attributes, FFF microstructure, and UV–temperature–moisture weathering on material and part durability. The scope is service durability, defined here as retention of application-relevant structure and properties during exposure. Industrial composting and biodegradation are considered only where they clarify the durability–degradability trade-off; they are not used as proxies for outdoor weathering [5,7,36].

The review addresses five interrelated research questions:

Q1. How do reprocessing and residual moisture affect PLA molar mass, rheology, crystallization, filament quality, and printability?

Q2. How do TiO_2_ phase, loading, surface chemistry, and dispersion influence the initial and weathered behaviour of virgin and reprocessed PLA?

Q3. How do FFF interfaces, raster orientation, infill, and connected voids affect environmental ingress, damage localization, and property retention?

Q4. Under what conditions can accelerated weathering results be validated against natural exposure to support durability assessment in tropical environments?

Q5. Which experimental controls, characterization methods, and reporting practices are needed to improve cross-study comparability and future service-life modelling?

The main contribution of this review is to bring together findings that are often examined separately and assess how reprocessing, TiO_2_ modification, printed architecture, and environmental exposure may collectively influence PLA durability. The analysis distinguishes experimentally observed effects from proposed mechanisms and identifies limitations that make direct comparisons between studies difficult. Based on these findings, the review proposes research priorities for improving experimental design, material characterization, reporting practices, and comparisons between accelerated weathering and natural outdoor exposure.

Figure 1 provides an overview of the review, illustrating the relationships between processing conditions, material characteristics, FFF architecture, weathering mechanisms, and durability assessment. It also identifies the main research gaps and priorities discussed in the following sections. The arrows indicate thematic relationships rather than experimentally established causal connections. Section numbers refer to the corresponding manuscript sections.

## 2. Review Methodology

This narrative review examines how PLA reprocessing, TiO_2_ characteristics, FFF architecture, and weathering jointly affect the durability of printed components. Literature was identified mainly through Semantic Scholar [47] and OpenAlex [48], complemented by the authors’ reference library, citation tracking, and targeted publisher or DOI checks, using the thematic search domains in Table 1. The main search was conducted in July 2026 and publications up to June 2026 were considered; later reference reconciliation updated the inventory without repeating the database search. Because feedstock, fabrication, exposure, and measured responses differed among studies, effect sizes were not pooled, and findings were compared according to the relationship each study can support.

### 2.1. Search Strategy and Record Management

Six complementary search domains were used to identify studies addressing individual factors and their intersections. Table 1 summarizes representative terms used across the bibliographic platforms, with spelling variants and search expressions adapted to each platform. Backward and forward citation checking and publisher records supported the identification and verification of publications relevant to specific claims.

The search identified 5298 records from four sources: Semantic Scholar (4896), OpenAlex (56), the authors’ RIS library (314), and citation or DOI checks (32). After removal of 1947 duplicates, 3351 unique records were archived. Four further records were added during reference reconciliation, giving an updated inventory of 3355 records (Figure 2). Prioritization was applied only to the 3351 archived records and was based on automated metadata screening: 2829 records were flagged as priority records for closer examination, and the remaining 522 were kept as background records for context. These two groups are organizational labels, not inclusion or exclusion decisions. The lead author then assessed sources against the criteria described in Section 2.2, and 145 sources (140 journal articles and 5 contextual references) were incorporated as evidence in this review.

### 2.2. Selection and Evidence Appraisal

The lead author selected sources from titles and abstracts and, when a PDF was available, from the full text, according to their relevance to the research questions (Q1–Q5), experimental transparency, environmental relevance, and comparability across studies. Preference was given to studies reporting formulations, processing history, controls, exposure protocols, and measured outcomes. When full text was unavailable, abstracts supported only the statements they substantiated, and no experimental details were inferred. Foundational publications on size-dependent and autocatalytic hydrolysis, photodegradation, thermo-mechanical processing, and interlayer bonding were retained to establish mechanisms [9,10,17,34,49,50,51,52,53]. Studies of non-printed specimens, other polymers, or biomedical conditions were used only for transferable mechanistic information, with their limits stated, and studies focused solely on biodegradation or composting [7,8,25,36] were not treated as evidence of outdoor durability. Publications after June 2026 and sources lacking sufficient information to support the intended claim were not used.

Each claim was assigned an evidence role: direct (D), a measured relationship in a relevant material or printed system; bridge (B), linking relevant domains but requiring transfer across material, processing, geometry, or exposure; and mechanistic (M), explaining a pathway without a quantitative result for the target component. These roles describe applicability, not experimental quality, and no numerical quality score or pooled estimate was assigned. The co-authors reviewed the interpretations and checked original publications when needed; no duplicate screening or agreement measure was carried out. Similar trends were not treated as equivalent when initial states or boundary conditions differed, and apparent counterexamples (retained strength despite recycling, reduced crystallinity after TiO_2_ addition, and accelerated damage in a rutile-containing composite [29,32,54]) were used to identify variables that could explain disagreement. Evidence on individual relationships does not establish the combined interaction of reprocessing, characterized TiO_2_, FFF architecture, and tropical weathering [29,30,32].

## 3. State of the Art

Existing reviews have addressed several aspects of PLA processing, modification, and environmental degradation, but these topics are often examined separately. Reviews on FFF discuss interlayer bonding, processing parameters, rheology, semicrystalline feedstocks, and void formation. Studies reviewing PLA processing and recycling describe the effects of melt degradation and repeated processing, while weathering reviews focus on photochemical and hydrolytic degradation mechanisms. Reviews of PLA–TiO_2_ composites, in turn, emphasize particle dispersion and the development of multifunctional properties [6,12,13,25,36,55,56,57,58]. Together, these publications provide a foundation for understanding the factors that influence PLA durability. However, their findings need to be considered in relation to reprocessed PLA containing well-characterized TiO_2_, manufactured into anisotropic components through FFF and subsequently exposed to environmental conditions. Table 2 compares the scope, limitations, and remaining research gaps of the selected reviews. Greater attention is given to publications addressing the relationships among PLA reprocessing, TiO_2_ modification, FFF, and environmental durability, followed by reviews that provide complementary information on degradation mechanisms and evaluation methods.

Recent publications further clarify the contribution of the present review. Pérez-Fonseca et al. [59] address PLA recycling and include weathering and degradation before recycling, while Sola and Trinchi [61] examine the recycling of composite feedstocks and printed components in FFF. Frigione and Rodríguez-Prieto [64] discuss the relationship between accelerated ageing and long-term material behavior, and Solek et al. [60] consider polymer degradation, additive manufacturing, and durability assessment standards. These studies demonstrate that recycling, additive manufacturing, and ageing have already been examined together in different contexts. Therefore, the contribution of the present review is not to establish a new connection between these topics, but to examine the specific combination of controlled PLA reprocessing, TiO_2_ characteristics, FFF architecture, and weathering under tropical environmental conditions.

As shown in Table 2, the present review addresses an intersection of topics rather than replacing or broadly extending the scope of existing reviews. Its contribution lies in examining how processing conditions establish the initial material and printed-component properties that may influence subsequent environmental degradation. It also distinguishes direct experimental evidence from findings obtained in related material systems and from proposed degradation mechanisms. This distinction is important because improvements in initial stiffness or thermal stability may occur alongside faster surface oxidation, reduced ductility, or changes in failure behavior after environmental exposure [29,30,32,46].

## 4. Processing-Dependent Initial State

### 4.1. Reprocessing Effects on PLA

PLA can degrade during extrusion through hydrolysis, thermal reactions, thermo-oxidation, transesterification, and mechanically assisted chain scission. The relative contribution of each mechanism depends on residual moisture, temperature, residence time, shear conditions, oxygen availability, catalyst residues, end groups, and stabilizers [6,18,19,20]. Because screw speed affects both shear and residence time, processing conditions cannot be compared reliably across studies without information on screw geometry, throughput, residence time, and melt temperature [6,20]. Differences in PLA grade, initial molar mass, stereochemical composition, and additive content can influence the response to subsequent processing. Similar extrusion conditions do not necessarily produce comparable levels of degradation when the starting materials differ.

Changes in PLA molar mass are commonly evaluated using size-exclusion chromatography, intrinsic viscosity, melt flow rate, or oscillatory rheology. Zero-shear viscosity is particularly sensitive to changes in molecular entanglement, but a single melt flow rate measurement cannot establish the degradation mechanism or molar-mass distribution [6,19,20]. Reprocessing studies frequently report reductions in viscosity or molar mass, although tensile strength may initially remain within experimental variability because it also depends on crystallinity, molecular orientation, defects, and testing conditions [17,22,54]. Therefore, the absence of a statistically significant reduction in tensile strength after one processing cycle does not necessarily indicate that the molecular structure remains unchanged. This distinction is important when comparing studies that rely on different characterization methods. A reduction in molar mass may be detected through rheological measurements before substantial changes become apparent in tensile properties. Moreover, differences in initial molar mass and crystallinity may affect the extent to which molecular degradation is reflected in mechanical performance.

Studies involving repeated extrusion further illustrate this distinction. Pillin et al. examined PLA subjected to up to seven thermomechanical processing cycles and reported progressive reductions in molar mass, rheological response, stress, and strain at break, while tensile modulus remained relatively stable [66]. Other studies involving multiple extrusion cycles, PLA grades, and processing equipment have similarly shown that apparent retention of mechanical properties may conceal chain scission, changes in crystallization behavior, and increasing sensitivity to processing conditions [22,49,67]. The differences in reported property retention may be partly explained by variations in processing temperature, residence time, and the initial characteristics of the PLA. Higher thermal exposure or longer residence times can increase the opportunity for chain scission, whereas differences in molecular structure and crystallization behavior may influence the resulting mechanical response. However, the relative contribution of these factors cannot always be established because processing conditions and initial material properties are not reported consistently across studies. The presence of layered minerals, residual catalysts, hydrolysis stabilizers, or chain extenders can further modify these effects. Consequently, the number of reprocessing cycles alone does not adequately describe the extent of material degradation [6,68,69,70].

However, the effects of recycling on mechanical properties are not consistently negative. Anderson reported a 10.9% reduction in tensile yield strength, a 6.8% increase in shear yield strength, a 2.4% reduction in hardness, and no statistically significant change in tensile modulus following one recycling procedure. Nevertheless, recycled filament exhibited greater variability, and nozzle clogging was observed [54]. Agbakoba et al. found comparable thermal stability in filaments incorporating waste from failed prints, whereas a formulation containing waste biocomposite exhibited an 11% reduction in degradation onset temperature. Specimens containing 50% waste PLA also showed a 29% reduction in tensile strength [71]. Farias et al. observed that PLA retained its mechanical properties more effectively than a PBAT/PLA blend over three simulated recycling cycles, while PLA crystallinity measured during the second heating increased from approximately 2% to 13% [22]. These studies illustrate that the apparent effects of recycling depend not only on processing history but also on material composition and the property being evaluated. For example, the relatively small changes in some mechanical properties reported by Anderson contrast with the tensile strength reduction observed by Agbakoba et al., although the differences in recycled feedstock and experimental procedures prevent a direct comparison. Similarly, the increase in crystallinity reported by Farias et al. suggests that structural changes during recycling may influence mechanical behavior independently of molar-mass reduction. These findings demonstrate the technical feasibility of PLA recycling but also show that the results depend on waste origin, material formulation, the number and severity of processing cycles, and the properties evaluated [49,71,72].

Studies examining property recovery and material reuse further demonstrate why recycled PLA should not be treated as a single material category. Beltrán et al. investigated simulated ageing, mechanical recycling, and strategies for restoring material properties [73]. Gonçalves et al. examined repeated extrusion [67], Salah et al. evaluated recycled PLA reinforced with nanoclay [74], Tănase et al. optimized the processing of recycled printed PLA [75], and Sepúlveda-Carter et al. investigated simulated industrial PLA waste for potential reuse in food-contact applications [76]. Differences in feedstock origin, additives, contamination, processing equipment, and intended applications limit direct comparisons between these studies. In particular, studies using controlled or simulated waste streams may not represent the variability in post-consumer or repeatedly processed PLA. Likewise, the use of reinforcing fillers, stabilizers, or optimized processing conditions can influence property retention, making it difficult to separate the effects of recycling from those of material modification. Their results support the feasibility of recycling under specific conditions rather than demonstrating that recycled PLA consistently retains the properties of virgin material. Feedstock origin, stabilization strategies, and the severity of previous melt-processing cycles should therefore be considered separately when interpreting recycling performance.

Recent investigations have begun to examine the relationship between recycling and UV exposure. Janutėnienė et al. compared virgin and recycled PLA, ABS, and PETG before and after UV exposure and reported increased brittleness in recycled materials, with reductions in elongation ranging from approximately 16% to 45% across the evaluated polymers [77]. Jirků et al. examined a sequence involving printing, UV degradation, recycling, and reprinting using neat PLA and a PLA composite containing 10 wt.% coffee grounds. Their results showed that both reprocessing and filler composition influenced material behavior after exposure [78]. These studies provide useful evidence connecting recycling and UV degradation. However, Janutėnienė et al. did not establish a complete factorial comparison of recycling and UV exposure, and neither study isolated the effects of TiO_2_ crystalline phase, surface coating, or photocatalytic activity. Their findings therefore cannot be generalized to all recycled PLA formulations. Furthermore, differences in material composition and exposure sequences make it difficult to determine whether the observed changes arise primarily from previous processing, UV degradation, or interactions between these factors.

An earlier primary study directly links reprocessing and hygrothermal ageing. Badia et al. investigated virgin PLA and materials subjected to up to three reprocessing cycles using compression-moulded specimens immersed in distilled water at 65, 70, and 75 °C [79]. After three cycles, saturation water uptake decreased by approximately 25% relative to virgin PLA, while diffusion coefficients declined with reprocessing but increased with ageing temperature. Nevertheless, hygrothermal exposure caused further molar-mass loss and deterioration of thermal and dynamic-mechanical performance. FTIR and DSC analyses also revealed accelerated crystallization of shortened chains with successive reprocessing cycles, whereas higher ageing temperatures slowed crystalline reorganization. These findings demonstrate that reduced water uptake does not necessarily indicate improved hydrolytic durability, although their transfer to outdoor FFF components remains limited by the non-printed geometry and above-Tg immersion conditions.

### 4.2. Filament Extrusion and FFF Architecture

Filament production introduces an additional melt-processing cycle and requires adequate dimensional control. Variations in filament diameter affect material delivery during printing, while insufficient melt strength, particle agglomeration, moisture, and contamination can cause filament breakage or nozzle clogging [16,49,54,72]. Reviews of material extrusion and rheological studies show that feedstock transport, pressure drop, die swell, thermal conditions, and viscoelastic recovery are closely related factors affecting printability [55,57,80,81]. Direct pellet extrusion can eliminate the filament-production step, but it introduces additional requirements for feed control and consistent material deposition. Therefore, results obtained using pellet-fed systems should not be considered directly equivalent to those from conventional filament-fed FFF [16,29].

Interlayer bonding develops through contact between deposited strands, neck growth, molecular diffusion, and re-entanglement while the polymer remains sufficiently mobile. Cooling rate, nozzle temperature, deposited material temperature, time above the relevant thermal transition, contact pressure, and polymer relaxation time influence this process [9,10,11,12,82]. Experimental and theoretical studies also indicate that extrusion can stretch and partially disentangle polymer chains, making interlayer bonding dependent on the recovery of molecular configuration rather than temperature alone [81,83,84]. Reprocessing may reduce melt viscosity and improve material flow or contact between deposited strands. However, excessive molar-mass reduction can decrease the molecular entanglements required for strong interlayer bonding. Thus, the same rheological change may improve deposition while reducing interlayer strength, depending on the extent of degradation and the processing conditions [6,11,83].

The assumption that FFF interlayer interfaces are always weaker than the original filament is not universally valid. Under carefully controlled conditions, PLA specimenshave achieved interlayer strengths comparable to those of bulk filament, although bead geometry, surface grooves, and localized deformation can still reduce toughness or strain at failure [85,86]. Studies involving controlled toolpaths have also distinguished the effects of bead orientation and part density from those of interlayer adhesion [87]. Subsequent environmental exposure may modify these relationships. Therefore, achieving high inter-layer strength before weathering does not establish that this strength will be retained after hydrolysis, oxidation, or additional processing of recycled feedstock.

Voids in FFF components may form between adjacent deposited strands, between layers, near external perimeters, or within partially filled regions. Their volume, shape, orientation, and connectivity depend on bead geometry, toolpath, layer height, temperature, printing speed, extrusion flow rate, infill configuration, crystallization, and build position [13,15,88,89]. Consequently, nominal infill percentage alone does not adequately describe the internal structure of printed components. When moisture penetration is an important aspect of the investigation, three-dimensional porosity and interconnected void networks should be characterized using methods such as micro-computed tomography or validated stereological analysis [13,45,90,91].

Moisture conditioning and printing orientation may also interact in determining mechanical performance. In a study involving 27 specimens, Algarni found that raster angle and moisture conditioning jointly influenced the mechanical response of FFF PLA [92]. However, the limited sample size, use of a single material system, and specific conditioning procedure restrict the general applicability of these findings. In particular, an apparent optimum under one conditioning procedure does not establish a universally beneficial moisture content. Nevertheless, the study highlights the importance of reporting both moisture-conditioning conditions and raster orientation when comparing mechanical properties across investigations.

### 4.3. TiO_2_ Dispersion and Initial Properties

The initial properties of PLA–TiO_2_ composites depend on particle dispersion and interfacial adhesion, rather than TiO_2_ concentration alone. Surface-modified TiO_2_ can improve compatibility with the polymer matrix compared with unmodified particles, while agglomerates may act as stress concentrators and produce uneven thermal and photochemical responses [23,25,26]. Therefore, studies should report TiO_2_ crystalline phase and surface treatment and characterize particle dispersion at different length scales. A single high-magnification SEM image is insufficient to establish representative particle dispersion throughout the material [25,32].

The preparation method also influences particle distribution and interactions with the polymer matrix. Surface functionalization, in situ polymerization, solution processing, conventional melt blending, and high-shear extrusion can produce different particle–polymer interfaces and degrees of agglomeration [93,94,95,96,97]. Luo et al. reported that lactic-acid-functionalized anatase improved dispersion and modified the rheological, crystalline, and mechanical properties of PLA composites. Zhuang et al. and Li et al. used in situ preparation methods to obtain nanocomposites with different structural characteristics [93,94,95]. These findings demonstrate that material preparation influences particle distribution and composite properties. However, the use of non-printed specimens and the absence of previous PLA reprocessing limit their direct applicability to reprocessed PLA–TiO_2_ components manufactured through FFF.

The influence of TiO_2_ on PLA crystallization is similarly dependent on material formulation and processing conditions. Although TiO_2_ has acted as a nucleating agent in several studies, the magnitude and direction of the observed changes depend on particle characteristics, dispersion, PLA stereochemistry, thermal analysis conditions, and cooling rate [98,99,100,101]. Improvements in thermal transitions or decomposition temperatures should therefore be interpreted as changes in initial material properties rather than evidence of improved environmental durability [97,101]. This distinction is particularly relevant to FFF, where crystallization can influence deposited strand geometry, shrinkage, interlayer bonding, and residual stresses before environmental exposure begins [55,88,89].

Bergaliyeva et al. investigated virgin PLA and blends containing 75 wt.% reprocessed PLA, with TiO_2_ incorporated at concentrations of 0, 3, and 7 wt.%, using direct pellet printing. The onset of thermal decomposition generally increased with TiO_2_ concentration. However, crystallinity decreased from approximately 15% in virgin PLA to 11% and 9% at 3 and 7 wt.% TiO_2_, respectively. In the reprocessed PLA blend, crystallinity decreased from approximately 20% to 15% and 10% at the corresponding concentrations [29]. TiO_2_ incorporation reduced tensile strength in specimens produced from virgin PLA but increased the horizontal tensile strength of the reprocessed blend from approximately 48.5 MPa to 52.5–53.6 MPa [29]. The reported crystallinity values follow are consistent with the data and discussion presented in the original study, although the abstract describes an increasing trend.. This inconsistency should be considered when interpreting the results. Overall, the findings demonstrate that the effects of TiO_2_ depend on the initial PLA formulation and should not be described as consistently reinforcing or weakening.

Rodríguez et al. examined six filament formulations containing virgin or reprocessed PLA with TiO_2_ concentrations of 0.25 and 0.5 wt.%. The study reported improvements in stiffness and thermal resistance in TiO_2_-containing materials and demonstrated the feasibility of producing printable composite filaments at relatively low particle concentrations [30]. Yilmaz investigated printing temperatures ranging from 180 to 240 °C and reported that TiO_2_ increased crystallinity from 6.4% to 17.4% and improved flowability by 9–29%. However, some mechanical properties decreased, although higher printing temperatures partially compensated for these reductions [31]. Together with the findings of Bergaliyeva et al., these studies indicate that the effects of TiO_2_ depend on particle concentration, PLA reprocessing, and printing temperature. Nevertheless, none of these investigations established the long-term weathering durability of printed components containing reprocessed PLA and TiO_2_ [29,30,31].

Table 3 compares six representative experimental studies according to material composition, manufacturing method, environmental exposure, principal findings, and limitations of applying their results to the present review. The comparison distinguishes initial material characterization from accelerated and natural weathering experiments and highlights why results obtained before exposure or from non-printed specimens cannot independently establish the durability of weathered FFF components. Table 4 provides a broader comparison of the available experimental evidence.

## 5. Multiscale Weathering Mechanisms

In this review, a degradation mechanism refers to the physical or chemical process responsible for changes in the material, while a material-state change refers to the measurable consequence of that process. A durability outcome describes how these changes affect the functional performance of a component. For example, hydrolytic chain scission is a degradation mechanism, a reduction in molar mass is a material-state change, and a loss of tensile strength or ductility is a durability outcome. These distinctions are important because structural changes, such as crystallization or annealing, may temporarily preserve certain mechanical properties even when molecular degradation is occurring.

### 5.1. Photolysis and Photo-Oxidation

PLA photodegradation is commonly associated with the excitation of carbonyl groups, followed by chain-scission reactions that may involve Norrish type I and type II pathways. These reactions can produce unsaturated structures, radicals, hydroperoxide-related species, and products with lower molar mass [24,34,35,36,38]. The relative importance of these pathways depends strongly on the wavelength of incident radiation. Although UV-C and UV-B experiments can provide information about photodegradation mechanisms, their results cannot be directly equated with exposure to natural sunlight because terrestrial solar radiation has a different spectral distribution and short-wavelength cutoff [28,34,39,42].

Early studies of polyester photolysis, quantitative UV-C experiments, artificial weathering investigations, and more recent molecular modelling have shown that wavelength, oxygen availability, specimen thickness, and initial molar mass influence degradation kinetics and the products formed [38,50,114,115,116]. Belbachir et al. demonstrated that photodegradation can be incorporated into a constitutive model describing changes in elastic–viscoplastic behavior. Santonja-Blasco et al. also showed that thermal, biological, and photochemical treatments produce different degradation and crystallization kinetics [117,118]. Although these studies provide useful information about the underlying mechanisms, most were conducted using films or moulded specimens and therefore do not fully represent oxygen and radiation gradients within the walls of FFF components. From a mechanical perspective, photochemical chain scission can reduce molar mass and molecular entanglement, potentially decreasing ductility and elongation at break. Surface oxidation and the development of microcracks may also promote premature failure by creating local stress concentrations. However, the resulting changes in tensile strength and modulus depend on the extent and distribution of degradation, as well as the initial crystallinity and morphology of the material [35,36,38].

ATR-FTIR spectroscopy is useful for monitoring changes in surface chemistry, but reliable interpretation requires consistent baseline correction, normalization, contact pressure, and definition of the indices used. Changes in the intensity or position of carbonyl-related absorption bands may result from overlapping reactions involving the formation and consumption of chemical groups. Consequently, these changes should not be interpreted as a direct or universal measure of chain scission throughout the material [35,36,46]. When molar-mass reduction is a central outcome, surface spectroscopy should be complemented by size-exclusion chromatography or a validated viscosity-based method [6,32].

Figure 3 combines pre-exposure characterization of as-printed specimens (FTIR and SEM) with the photolysis and photo-oxidation sequence discussed in this subsection.

### 5.2. Water Transport and Autocatalytic Hydrolysis

Water can cleave PLA chains through hydrolysis, producing hydroxyl- and carboxyl-terminated groups. Hydrolysis generally proceeds more readily in accessible amorphous regions and is influenced by temperature, specimen thickness, crystallinity, morphology, chain-end chemistry, and the transport and retention of acidic oligomers [5,119,120]. In FFF components, voids and interfaces between deposited strands modify the available surface area and may provide connected pathways for water penetration. Therefore, water uptake measured in dense films cannot be directly applied to printed components with different internal architectures [13,45,46].

Previous studies have established that PLA hydrolysis can depend on specimen dimensions and may become autocatalytic when acidic degradation products accumulate within the material [121,122]. Molar mass, D-lactide content, crystalline structure, and initial morphology also influence the progression of degradation [51,52,53,120]. Accordingly, exposure to humid air, condensation, and liquid-water immersion should be treated as different experimental conditions. Even at similar temperatures, differences in water activity and the transport of degradation products can produce different material responses [123,124,125].

Studies involving printed components confirm that internal architecture affects moisture penetration and degradation, although they do not establish a universal degradation rate. Kakanuru and Pochiraju reported temperature- and material-dependent water penetration and localized deformation in additively manufactured specimens. Menezes et al. modelled the effects of prolonged marine exposure, while Klimczuk et al. related severe hydrolytic degradation to changes in the architecture and molecular structure of printed components [90,91,109]. Sabalina et al. identified stages involving diffusion, degradation, and leaching in printed polyester blends, whereas Moreno Nieto et al. demonstrated that discontinuities associated with additive manufacturing can increase water uptake beyond values expected from bulk-material properties [108,112]. Differences in temperature, water composition, polymer formulation, and specimen architecture prevent direct quantitative comparisons. Nevertheless, these studies support the need to characterize void connectivity and wall thickness when investigating moisture transport.

Bergaliyeva et al. compared thermal and hydrothermal accelerated ageing of printed PLA and observed a reduction in tensile strength after prolonged hydrothermal exposure, including measurements at 1344 h [102]. Ekinci et al. investigated thin, single-layer FFF films exposed to hydrolytic conditions at 37 °C for up to 280 days. They reported reductions of approximately 60% in modulus, 86% in ultimate tensile strength, and 80% in molecular weight, while crystallinity increased by approximately 143% [103]. These results demonstrate that substantial molecular and mechanical changes can occur alongside increasing crystallinity. However, the magnitude of these changes depends on specimen geometry and exposure conditions, and the results should not be interpreted as predictions of outdoor durability in tropical environments. Similarly, converting accelerated hydrothermal exposure times into years of outdoor use requires evidence that the relevant degradation mechanisms are comparable under both conditions [102,103]. The results of Ekinci et al. [103] illustrate how hydrolytic chain scission can be accompanied by substantial reductions in molar mass and mechanical performance, even when crystallinity increases. Although shorter polymer chains may crystallize more readily, the resulting structural changes do not necessarily compensate for the loss of molecular connectivity. Consequently, tensile strength, modulus, and elongation at break may respond differently depending on the extent of hydrolysis, crystalline morphology, and specimen geometry. In printed components, moisture-induced degradation at interlayer interfaces may also affect failure behavior by weakening regions where stresses are transferred between deposited strands.

The sequence of processing and environmental exposure also influences the final material properties. Souissi et al. exposed PLA filament to UV radiation before printing and subsequently examined the effects of raster orientation on mechanical properties and water absorption [106]. Their experimental approach demonstrates that degradation occurring before deposition can influence both the filament entering the nozzle and the resulting printed component. However, exposing filament before printing is not equivalent to weathering a finished FFF component. The findings therefore provide supporting evidence for the effects of pre-printing degradation rather than direct evidence of outdoor durability.

Reprocessing may increase susceptibility to hydrolysis by reducing molar mass and increasing the concentration of reactive chain ends. However, an increase in crystallinity may restrict water penetration into more ordered regions of the polymer [19,22,119,120]. These competing effects suggest that the relationship between reprocessing and hydrolytic degradation may not be linear. Measurements limited to mass change or crystallinity alone are insufficient to determine the relative contribution of these mechanisms.

Figure 4 summarizes the sequence from water access to hydrolysis and autocatalysis, and the resulting changes in material state and durability.

### 5.3. Thermal Ageing and Annealing

Temperature influences both chemical reaction rates and molecular mobility in PLA. Exposure near or above the glass transition temperature can promote structural relaxation, cold crystallization, bonding between adjacent deposited strands, and relaxation of residual stresses. At the same time, elevated temperatures can accelerate hydrolysis and oxidation [4,33,44,102]. Consequently, annealing may reduce void content or improve the short-term retention of certain properties while molecular degradation, dimensional changes, or embrittlement develop under other exposure conditions [46,111]. Conclusions based on a single mechanical property measured at the end of an exposure period may therefore differ from those obtained using additional properties or intermediate measurements.

Faizaan et al. reported increasing crystallinity accompanied by decreasing tensile strength during prolonged exposure to UV-B radiation and humidity. In contrast, Tejedor et al. found that controlled annealing reduced void content and water uptake and limited the deterioration of flexural and dynamic-mechanical properties during salt-water ageing [46,111]. Weathering studies involving PLA composites reinforced with clay, halloysite, and glass fibres have also shown that modulus, strength, toughness, molar mass, and crystallinity do not necessarily change at the same rate or in the same direction [126,127,128]. These findings are not necessarily contradictory because differences in radiation spectrum, temperature, formulation, and exposure duration may favor different combinations of structural relaxation, crystallization, chain scission, embrittlement, and crack propagation. These competing processes help explain why changes in crystallinity do not necessarily produce proportional changes in mechanical properties. Increased crystallinity may contribute to stiffness retention, while chain scission, reduced molecular connectivity, or the development of defects can simultaneously reduce tensile strength and elongation at break. Similarly, annealing may improve interlayer bonding or reduce internal defects without necessarily preventing subsequent chemical degradation. Therefore, modulus retention alone should not be considered sufficient evidence of improved durability, particularly when ductility and failure behavior are not evaluated [46,111,126,127,128].

Studies examining multiple environmental factors provide further evidence of these interactions. Jiang et al. identified combined effects of light, temperature, and humidity in non-printed PLA, while Goetjes et al. distinguished hydrolytic degradation from thermo-oxidation under warm and humid conditions [125,129]. Although neither study directly represents a printed component exposed to tropical weathering, both demonstrate the difficulty of attributing material changes to a single environmental factor when several stresses are applied simultaneously.

Figure 5 shows the thermal transitions and thermal stability of as-printed specimens that frame the structural and chemical consequences of thermal exposure.

### 5.4. TiO_2_: UV Shielding and Photocatalysis

TiO_2_ can reduce the transmission of incident radiation through absorption and scattering while also generating reactive species at photocatalytically active surfaces. The relative importance of these effects depends on crystalline phase, surface coating, particle dispersion, concentration, specimen thickness, and the spectral characteristics of the radiation [24,25,28,32]. Surface modification may improve particle dispersion and adhesion to the polymer matrix, but it can also alter the accessibility of photocatalytically active sites. Therefore, describing TiO_2_ simply as surface-modified is insufficient without specifying the type of modification and its potential effects [23,26].

Experimental studies on PLA–TiO_2_ composites illustrate the competing effects of radiation attenuation and photocatalytic degradation. Some transparent nanocomposite films have been developed specifically to promote photodegradation, while other investigations have reported accelerated photochemical or hydrolytic degradation when TiO_2_ surfaces remain photocatalytically active [98,130,131,132]. Conversely, approaches involving polyurethane toughening, organic UV absorbers, particle-size selection, or modifications to the particle–polymer interface have improved selected measures of property retention after UV exposure [133,134,135]. These findings indicate that TiO_2_ cannot be classified universally as either a stabilizing or degradation-promoting additive. Its overall effect depends on the balance among radiation attenuation, reactive-species formation, polymer mobility, and resistance to crack development, as well as the properties used to evaluate durability. These competing effects may also lead to different mechanical responses. Radiation attenuation can limit photochemical changes in the polymer matrix, potentially contributing to the retention of tensile properties. Conversely, photocatalytic reactions near exposed TiO_2_ particles may promote localized chain scission and surface damage, increasing susceptibility to crack initiation and premature failure. Particle agglomeration and poor interfacial adhesion may further influence stress transfer and fracture behavior. Consequently, changes in tensile strength, modulus, or elongation at break cannot be attributed solely to the UV-shielding or photocatalytic activity of TiO_2_ without considering particle dispersion, initial material properties, and processing history [24,32,93,133,134,135].

Hydrolytic studies introduce additional considerations. TiO_2_ has been shown to modify the long-term degradation of PLA under aqueous conditions, although the response depends on the formulation. Reinforcing particles with different chemical characteristics can also produce different responses to water exposure [99,130,136]. Studies involving composting or biodegradation have further demonstrated that functionalized TiO_2_ can influence disintegration rates. However, these findings concern end-of-life degradation under specific conditions and should not be interpreted as direct evidence of outdoor durability [137,138].

Antunes et al. investigated compression-moulded PLA containing 3 wt.% rutile TiO_2_ under accelerated exposure involving UVA-340 radiation and condensation. The TiO_2_-containing composite exhibited earlier surface damage and crystallization than neat PLA [32]. However, the composite also had a substantially lower initial molar mass than the neat PLA control. Consequently, the accelerated degradation cannot be attributed exclusively to TiO_2_ without considering differences introduced during material preparation [32]. This example highlights the importance of comparing TiO_2_-containing formulations with neat PLA controls subjected to identical processing conditions. Additional comparisons using optically comparable inert particles and coated and uncoated TiO_2_ could help distinguish radiation attenuation from photocatalytic effects [93,133,134].

Figure 6 summarizes the competing UV-shielding and photocatalytic pathways of TiO_2_ and the controls needed to separate them.

### 5.5. Architecture–Degradation Feedback

The relationships discussed in this review suggest that processing, material structure, and environmental degradation may influence one another. Reprocessing changes PLA molar mass and rheological behavior, while these properties, together with printing conditions, affect deposited strand geometry and interlayer bonding. The resulting architecture influences moisture penetration and the distribution of mechanical stresses. During weathering, chemical degradation and changes in crystallinity may promote cracking and other forms of damage, potentially creating additional pathways for environmental exposure and increasing local stress concentrations [6,12,13,32]. TiO_2_ distribution may further influence these processes by modifying radiation attenuation or promoting photocatalytic reactions in specific regions of the component.

These relationships remain a proposed explanation rather than a universally established sequence of effects. In particular, particle dispersion and the location of agglomerates need to be characterized experimentally because nominal TiO_2_ concentration does not indicate whether particles are concentrated near exposed surfaces, within deposited strands, or at interlayer interfaces [26,32,93]. Figure 7 summarizes the proposed relationships among reprocessing conditions, TiO_2_ characteristics, FFF architecture, environmental exposure, changes in material structure, and retained properties. The connections shown in the figure represent hypotheses that can be evaluated experimentally rather than established directions or magnitudes of individual effects.

Under natural outdoor exposure, photodegradation, hydrolysis, and thermal ageing may occur simultaneously and influence one another. UV radiation can induce chain scission and surface changes, potentially affecting subsequent moisture penetration and oxidation. At the same time, absorbed water can promote hydrolytic degradation, while elevated temperatures may accelerate chemical reactions and modify molecular mobility and crystallization behavior. These processes may produce competing effects, as increased crystallinity can restrict water penetration into ordered regions even when molecular degradation continues. The combined influence of environmental factors is supported by studies involving UV radiation and humidity [46], light, temperature, and moisture [129], and hydrolytic and thermo-oxidative conditions [125]. However, differences in materials and exposure protocols make it difficult to determine the individual contribution of each mechanism or establish whether their combined effects are additive or synergistic.

Repeated wetting and drying introduce further complexity because moisture penetration, retention, and evaporation may vary throughout the exposure period. Temperature fluctuations can also modify these processes, particularly in FFF components where voids, interlayer interfaces, and wall thickness influence moisture transport. As degradation progresses, surface damage and cracking may create additional pathways for water and oxygen, potentially accelerating local deterioration. Conversely, structural changes such as crystallization or annealing may temporarily limit moisture penetration or preserve certain mechanical properties [13,45,46,111]. These competing processes suggest that the durability of printed PLA cannot be reliably predicted from isolated UV, hydrothermal, or thermal ageing tests. Although accelerated studies combining UVA radiation and condensation provide useful evidence [32], further investigations involving controlled environmental cycles and natural outdoor exposure are needed to determine how these mechanisms interact under realistic conditions.

The relationship between degradation mechanisms and mechanical performance is therefore not straightforward. Reductions in molar mass may decrease molecular entanglement and ductility, while changes in crystallinity can influence stiffness and strength in different ways. Chemical modifications and morphological damage, including surface cracks, voids, and weakened interlayer interfaces, may further affect stress distribution and failure behavior. As a result, tensile strength, modulus, and elongation at break may evolve differently during weathering, and the retention of one property does not necessarily indicate that the material remains structurally unchanged. For FFF components, these relationships are particularly important because failure may be influenced by both molecular degradation and the location of defects within the printed architecture [12,13,32,46,103,111].

## 6. Interpreting Durability Differences

### 6.1. Areas of Convergence

Five recurring findings provide a basis for comparing the available studies, although their magnitude and applicability depend on the materials and experimental conditions evaluated. First, controlling moisture before each melt-processing step is important because hydrolysis during processing can reduce PLA molar mass and viscosity [6,20,66]. Second, the number of recycling cycles alone is insufficient to characterize processing-induced degradation unless temperature, shear conditions, residence time, and drying procedures are also reported [6,21,22,72]. Third, FFF interlayer interfaces and voids influence mechanical anisotropy and may provide pathways for moisture penetration and localized deformation, although properly formed interlayer bonds can approach bulk-material strength before environmental exposure [12,13,85,90]. Fourth, the combined effects of UV radiation, temperature, and moisture can accelerate PLA weathering or change the degradation processes involved [33,44,46,129]. Finally, the influence of TiO2 depends on material formulation and exposure conditions, rather than being consistently beneficial or detrimental to durability [24,32,98,133,134].

### 6.2. Apparent Contradictions

#### 6.2.1. Crystallinity and Chain Scission

Reprocessing and weathering can increase PLA crystallinity as shorter or more mobile polymer chains reorganize. However, some PLA–TiO_2_ formulations exhibit lower crystallinity with increasing particle concentration [19,22,29,46]. Annealing can also increase crystallinity while reducing void content and water uptake, whereas crystallization associated with molecular degradation does not necessarily preserve toughness [111,138]. Crystal structure introduces another variable because different crystalline forms of PLLA exhibit different hydrolytic degradation behavior [53]. Consequently, studies should distinguish crystallinity measured during the first and second DSC heating cycles, complement thermal analysis with diffraction measurements, and specify whether TiO_2_ mass was excluded when calculating crystallinity relative to the PLA fraction.

#### 6.2.2. Mechanical Property Retention

Recycled PLA may retain tensile strength comparable to virgin material after limited processing, while other recycled formulations exhibit substantial reductions [22,54,71]. Similarly, TiO2 incorporation can increase tensile strength in a reprocessed PLA blend while reducing it in a virgin PLA formulation [29]. Environmental exposure may decrease tensile strength, whereas controlled annealing can limit certain losses during hydrothermal ageing by modifying crystallinity and void structure [46,111]. These findings demonstrate that mechanical strength depends on several interacting material and structural characteristics. Therefore, tensile strength alone cannot adequately describe degradation without complementary measurements of molar mass, ductility, fracture location, and defects.

#### 6.2.3. TiO_2_: Thermal vs. UV Stability

Improvements in the initial thermal stability of PLA–TiO_2_ composites do not necessarily indicate greater resistance to photodegradation [29,30]. For example, the temperature corresponding to 5% mass loss during thermogravimetric analysis under nitrogen or air reflects a different degradation process from the reactions occurring at polymer surfaces exposed to UV radiation and moisture. Similarly, describing a composite as UV resistant based exclusively on radiation attenuation, color retention, or thermogravimetric results does not establish the same level of durability as measurements of molar-mass retention and mechanical performance after exposure [25,32].

#### 6.2.4. Filler Effects by Exposure Type

Montmorillonite improved selected mechanical properties retained after one accelerated weathering procedure, whereas Cloisite 30B promoted photo-oxidation during natural outdoor exposure [126,139]. These results are not necessarily contradictory because the studies differ in filler characteristics, dispersion, radiation spectrum, moisture conditions, and measured properties. Reinforcing particles may help preserve certain mechanical properties even when molecular degradation increases. Accordingly, mechanical property retention and chemical stability should be evaluated independently.

#### 6.2.5. Interlayer Strength and Part Anisotropy

Under controlled conditions, interlayer bonds in unweathered PLA components can achieve strengths comparable to those of the original filament. Nevertheless, deposited strand geometry, surface grooves, raster orientation, voids, and localized deformation may still reduce toughness and overall component performance [85,86,87]. Weathering introduces additional changes in polymer chemistry and crack development that can further influence mechanical behavior [45,90]. Therefore, weak interlayer bonding should not be inferred from printing orientation alone. Interlayer fracture behavior, effective load-bearing cross-section, and defect distribution should be characterized to distinguish bonding limitations from geometric and environmental effects.

Table 5 summarizes the main apparent contradictions identified in the literature, the variables that may explain them, and the measurements needed to evaluate these explanations. The proposed interpretations should be treated as hypotheses requiring experimental verification rather than definitive explanations for every difference between studies.

### 6.3. Limits of Cross-Study Inference

The available evidence indicates that changes in molecular structure, crystallinity, and printed-component geometry may compensate for one another under specific testing conditions without preserving all aspects of durability. For example, the recycled PLA specimens investigated by Anderson, the TiO_2_-containing blends examined by Bergaliyeva et al., and the weathered composite sheets studied by Antunes et al. differed in their initial material characteristics, processing conditions, and measured properties [29,32,54]. Their results therefore cannot be compared using a single measure of improvement or degradation. Instead, these studies help identify the experimental controls needed to distinguish the effects of material composition from those of processing and environmental exposure.

These limitations also affect the interpretation of interactions among reprocessing, TiO_2_ incorporation, FFF architecture, and weathering. Separate studies reporting recycling-induced degradation, improvements associated with fillers, or embrittlement during environmental exposure provide a basis for proposing interactions, but they do not establish whether these factors produce synergistic or antagonistic effects within the same printed formulation. Such conclusions require controlled factorial experiments in which the initial material characteristics and processing conditions are comparable. The research priorities proposed in the following sections therefore arise from the limitations identified in the available evidence, rather than from the number of publications reporting a particular result or the frequency of favorable findings.

## 7. Tropical Weathering and Validation

In this review, direct evidence of durability under tropical conditions refers to results obtained from printed PLA-based specimens exposed to natural tropical environments with adequately documented exposure conditions, or from accelerated ageing experiments independently validated against comparable tropical field data. Relevant environmental variables include solar or UV radiant exposure, air and specimen-surface temperature, relative humidity, rainfall or time of wetness, specimen orientation, and exposure duration. Laboratory studies combining UV radiation, heat, and moisture without corresponding field validation provide supporting evidence for assessing their relevance to tropical environments. In contrast, single-factor experiments, severe immersion conditions, extreme pH or temperature, short-wavelength irradiation, and studies involving substantially different materials or geometries are primarily useful for understanding degradation mechanisms. These distinctions indicate how the findings can be interpreted and applied, rather than the methodological quality of the individual studies.

### 7.1. Limits of Chamber-Hour Comparisons

ISO 4892-1 emphasizes the importance of considering the radiation source and operating conditions when interpreting artificial weathering results, while ASTM G154 requires the specific exposure cycle to be reported for meaningful comparison [39,41]. Equivalent exposure durations under UVA-340, UV-B, xenon-arc radiation, dry heat, or natural outdoor conditions do not necessarily produce comparable degradation. These methods differ in spectral distribution, radiant exposure, specimen-surface temperature, and moisture or wetting conditions [40,41,42]. Therefore, comparisons between studies should consider the actual radiation and environmental conditions rather than elapsed exposure time alone.

Natural outdoor exposure is essential for evaluating durability under tropical conditions because local solar radiation, temperature, rainfall, wetting duration, atmospheric pollutants, and specimen orientation influence material degradation. ASTM G7/G7M recognizes that relative durability may vary between exposure locations and that results from a single site cannot establish a universally applicable degradation rate [43]. Chopra et al. provide relevant evidence from the natural weathering of printed PLA. However, their findings from a specific location in India and a particular material formulation cannot be directly applied to Panama or other tropical regions without comparable meteorological and radiometric information [43,45].

Existing natural-weathering studies illustrate both the value and limitations of outdoor exposure data. Yew et al. investigated PLA composites under warm and humid conditions in Penang, Malaysia, while Zaidi et al. examined PLA/clay nanocomposites at another outdoor location using molecular, thermal, and mechanical characterization [139,140]. Both studies demonstrate that material formulation influences degradation under combined environmental conditions. However, the available information does not provide the complete spectral radiant exposure, specimen-surface temperature, and time-of-wetness records needed to establish direct equivalence with other tropical locations. These studies therefore provide useful examples of natural-weathering behavior, but they do not establish universal acceleration factors for tropical environments.

### 7.2. Two-Stage Tropical Validation

A suitable approach for evaluating the durability of printed PLA-based components under tropical conditions combines controlled accelerated-weathering experiments with natural outdoor exposure. The laboratory stage should include UV-only, moisture-only, thermal-only, and combined exposure conditions, with radiant exposure matched where applicable or explicitly documented. This approach allows the effects of individual environmental factors and their interactions to be investigated while helping distinguish structural changes associated with annealing from chemical degradation [33,44,102,129].

The outdoor stage should record global and UV irradiance, air and specimen-surface temperature, relative humidity, rainfall, time of wetness, specimen orientation, and atmospheric pollutants or salt deposition when relevant [39,43]. The same reference materials and printed formulations should be evaluated under laboratory and natural exposure conditions. This would allow relationships between accelerated and outdoor degradation to be established using comparable measured responses rather than assumed equivalence based on exposure duration.

UVA-340 fluorescent lamps provide controlled irradiation in the short-wavelength region of the terrestrial solar spectrum, whereas appropriately filtered xenon-arc systems can reproduce a broader portion of the solar spectrum [40,41,42]. UV-B exposure may accelerate certain degradation processes, but its spectral characteristics can differ substantially from natural sunlight. Consequently, results obtained under UV-B radiation should not be converted directly into outdoor exposure times without independent validation [46,105]. Similarly, condensation cycles provide controlled and potentially severe surface wetting but do not reproduce all the effects of rainfall, salt deposition, atmospheric pollutants, or biological activity [41,141]. Table 6 summarizes the principal advantages, limitations, reporting requirements, and appropriate interpretations of the different exposure methods.

### 7.3. Key Tropical Exposure Variables

Studies intended to evaluate durability under tropical conditions should, at minimum, report total and spectral radiant exposure, specimen-surface temperature, relative humidity, the duration of exposure above relevant temperature thresholds, wetting and drying periods, water chemistry, and specimen orientation. UV dose alone is insufficient to characterize the exposure because hydrolysis and structural changes associated with annealing also depend on moisture availability and temperature over time [33,39,119,120]. Likewise, average relative humidity does not adequately describe condensation or rainfall events that may introduce liquid water into interfaces between deposited strands and interconnected voids [13,41].

### 7.4. Criteria for Service-Life Inference

Calibration, validation, and prediction represent distinct stages in the development and application of durability models. Calibration involves estimating model parameters from an experimental dataset. Validation evaluates the model using independent observations that were not included in parameter estimation, preferably obtained under conditions representative of the intended application or through natural outdoor exposure. Prediction involves applying the validated model to estimate future material behavior or the time required to reach a defined failure criterion. Agreement between a model and the data used for calibration does not constitute independent validation. Similarly, a correlation between accelerated and natural exposure results does not, by itself, establish a reliable prediction of component lifetime.

An Arrhenius-type relationship may be expressed as k(T) = A exp[−Ea/(RT)], where k is the reaction-rate constant, A is the pre-exponential factor, Ea is the apparent activation energy, R is the universal gas constant, and T is the absolute temperature. However, obtaining a linear relationship in an Arrhenius plot does not demonstrate that the same degradation mechanism operates under accelerated and natural exposure conditions. Competing reactions, diffusion limitations, physical transitions, and changes in crystallinity may produce deviations from Arrhenius behavior [143,144]. Reliable extrapolation requires evidence that degradation products and failure mechanisms remain comparable across the evaluated temperatures, that relevant thermal transitions or crystallization changes do not invalidate the model assumptions, and that transport effects have been adequately considered. Predictions should also be evaluated using independent observations obtained at lower temperatures or under natural exposure conditions [109,124].

Menezes et al. and Deroiné et al. provide useful examples of accelerated degradation analysis under aqueous conditions, but their experiments do not reproduce the complete combination of solar radiation, wetting and drying, and mechanical conditions experienced by printed components in tropical environments [109,124]. Koike et al. developed a kinetic Monte Carlo approach to describe changes in molecular-weight distribution during hydrolysis. However, applying this model to printed PLA–TiO_2_ components would require additional consideration of moisture transport through FFF structures, TiO_2_-related reactions, fracture development, and validation against outdoor measurements [145]. Accordingly, durability predictions should report parameter uncertainty, specimen variability, and a failure criterion appropriate to the intended application rather than presenting a single deterministic lifetime.

## 8. Multiscale Durability Assessment

### 8.1. Baseline Material and Part State

Weathering results can be interpreted reliably only when the initial, unexposed material and component characteristics are adequately documented. At the material level, baseline characterization should include moisture content, molar-mass distribution, melt rheology or validated viscosity measurements, thermal transitions, crystallinity, and TiO_2_ crystalline phase and dispersion. At the printed-component level, relevant measurements include density or void fraction, connected porosity where feasible, deposited strand and layer dimensions, build orientation, surface roughness, and mechanical properties together with failure location [6,13,29]. Without this information, differences attributed to environmental exposure may instead originate from variations in compounding, filament extrusion, or printing conditions.

The study by Antunes et al. illustrates the importance of establishing comparable initial conditions. The PLA–TiO_2_ composite had a lower initial molar mass than the neat PLA specimen and underwent an additional melt-mixing step [32]. Although the observed surface damage demonstrates that the tested composite was susceptible to degradation under the applied weathering conditions, the results do not independently establish the contribution of TiO_2_. Including a neat PLA control subjected to identical processing conditions, together with an inert-particle or surface-coated TiO_2_ control, would help distinguish the effects of particle incorporation from those of processing-induced changes [24,25,32].

In this review, state variables refer to measurable characteristics of the material or printed component at a specific stage of processing or environmental exposure. These include molar-mass distribution, rheological properties, crystallinity and crystalline structure, TiO_2_ dispersion and surface characteristics, moisture content, connected porosity, interlayer geometry, residual stresses, oxidation state, and crack density. State variables differ from exposure variables, such as irradiance, temperature, and wetting conditions, and from durability outcomes, such as retained strength, ductility, dimensional stability, or functional failure. Measuring these characteristics consistently during feedstock preparation, filament production, printing, and weathering helps distinguish degradation mechanisms from their consequences and provides the information needed to develop predictive durability models.

### 8.2. Molecular and Chemical Analysis

Size-exclusion chromatography provides information about molar-mass distribution and dispersity, while rheological measurements help relate molecular characteristics to melt behavior and processability. Ideally, these measurements should be performed after compounding, filament production, and printing, as well as at selected intervals during environmental exposure. This approach allows changes associated with individual processing and weathering stages to be distinguished [6,20]. Melt flow rate can be used as a practical screening measurement, but it should not replace oscillatory rheology or direct molar-mass characterization when chain scission is a principal degradation mechanism [6,19].

Average molar mass alone may conceal important changes in the shape of the molecular-weight distribution. Hydrolysis models based on population-balance approaches or kinetic Monte Carlo methods can describe random chain scission, effects associated with polymer surfaces or crystalline regions, and changes in molecular-weight distributions over time [52,145]. These models should be evaluated using measurements obtained at multiple exposure intervals rather than a single final viscosity value. Their application to FFF components also requires consideration of water transport and specimen geometry.

ATR-FTIR, Raman spectroscopy, X-ray photoelectron spectroscopy, and UV–visible spectroscopy provide complementary information about changes in surface chemistry and optical properties. These techniques can help identify oxidation, unsaturated structures, hydroxyl formation, changes associated with TiO_2_, and radiation attenuation. However, differences in measurement depth and overlapping spectral bands require consistent acquisition conditions, normalization procedures, and clearly defined analytical indices [32,35,46]. When conclusions concern the integrity of an entire printed component, surface-sensitive measurements should be supported by complementary bulk characterization.

### 8.3. Thermal and Crystalline Analysis

For PLA composites, the degree of crystallinity obtained from differential scanning calorimetry (DSC) can be calculated as:

(1) $$X_{c}\left(\%\right)=\frac{\left(\Delta H_{m}-\Delta H_{cc}\right)}{\left(w_{\left(PLA\right)}\Delta H_{m}^{0}\right)}\times 100$$

where Δ*H_m_* is melting enthalpy, Δ*H_cc_* is cold-crystallization enthalpy, *w*_(*PLA*)_ is the PLA mass fraction, and $\Delta H_{m}^{0}$ is the explicitly selected reference enthalpy for fully crystalline PLA. The reference enthalpy, heating procedure, baseline correction, and treatment of multiple melting peaks should be reported because these choices affect the calculated crystallinity [4,29,46].

The first DSC heating cycle provides information about the thermal and crystalline state resulting from manufacturing and environmental exposure. In contrast, the second heating cycle partly removes the influence of the previous thermal conditions and reflects crystallization under the programmed cooling and reheating procedure. Both measurements are useful, but they describe different aspects of material behavior and should not be interpreted interchangeably [22,29]. X-ray diffraction (XRD) or wide-angle X-ray scattering (WAXS) can provide complementary evidence of changes in crystalline structure. Additional scattering or imaging techniques may be necessary to distinguish changes in polymer morphology from scattering associated with TiO_2_ particles [32,46].

### 8.4. FFF Architecture and Damage Analysis

Scanning electron microscopy (SEM) provides detailed information about local surface morphology and fracture characteristics, but observations from selected regions may not represent the entire printed component. Micro-computed tomography (µCT) allows three-dimensional characterization of void fraction, pore dimensions, orientation, and connectivity, making it particularly useful for investigating the relationship between FFF architecture and environmental penetration [13]. Repeated measurements or comparisons between equivalent specimens collected at different exposure intervals can help determine whether weathering-induced cracks connect previously isolated voids. TiO_2_ dispersion should be characterized separately from FFF porosity using representative image analysis, elemental mapping, or tomography at a resolution appropriate to the particle dimensions [25,26].

Mechanical characterization should include tensile strength, modulus, elongation at break, toughness or work to fracture, and failure location. Tensile strength alone cannot distinguish molecular stability from changes associated with densification, crystallization, or internal defects. Consequently, mechanical property retention should be interpreted alongside measurements of molar mass, ductility, and structural changes [46,111]. Fracture toughness, interlayer-specific testing, fatigue, and creep are particularly relevant to the long-term performance of functional printed components, although these properties are reported less frequently than conventional tensile strength [12,13].

When time-dependent constitutive models are developed, changes in damage parameters should be related to measured chemical or structural characteristics rather than fitted exclusively to a single stress–strain curve. Belbachir et al. incorporated photodegradation into a model of the elastic–viscoplastic behavior of PLA films, demonstrating a useful approach for describing changes in mechanical response [117]. However, extending this approach to FFF components requires additional consideration of printed architecture. Digital image correlation, fracture-location analysis, and repeated *µ*CT measurements could provide the spatial information needed to evaluate such models [90,91].

### 8.5. Exposure and Durability Metrics

Spectral radiant exposure should be determined from measured irradiance rather than estimated from exposure duration alone. It can be expressed as:

(2) $$H_{\left(\lambda_{1}-\lambda_{2}\right)}=\int_{0}^{t}\int_{\left(\lambda_{1}\right)}^{\left(\lambda_{2}\right)}E_{\lambda }\left(\tau \right)d\lambda d\tau$$

where *E_λ_* is spectral irradiance (W m^−2^ nm^−1^ when wavelength is expressed in nm) and *H*_(*λ*1−*λ*2)_ is radiant exposure (J m^−2^) over the stated wavelength interval, with time expressed in seconds. The radiation source, filters, calibration procedure, control wavelength, and integration range should be reported to allow comparisons between exposure conditions [39,40,41,42].

For a measured property (*P*), retained performance after exposure time (*t*) can be expressed as:

(3) $$R_{P}\left(t\right)=\frac{\;P\left(t\right)}{P\left(0\right)}\times 100\%.$$

where P(t) is the property measured after exposure and P(0) is its initial value. This normalization facilitates comparisons within a study but does not eliminate differences in initial material characteristics or testing procedures. Therefore, absolute property values, measurement uncertainty, sample size, conditioning conditions, and failure characteristics should also be reported [41,141].

In factorial experiments, interactions between two controlled factors can be evaluated by comparing the combined response with the sum of the individual responses. For factors *A* and *B*, an interaction contrast based on changes in a measured property can be written as:

(4) $$I_{AB}=\Delta P_{AB}-\Delta P_{A}-\Delta P_{B}+\Delta P_{0},$$

where *A* and *B* are controlled factors, *AB* is their combined condition, and 0 is the reference. Here, $\Delta P_{X}$ = $P_{X}\left(t\right)\;$− $P_{X}(0)$ denotes the change for condition *X* over a common interval, expressed on the same absolute response scale.

This contrast measures departure from additivity on the selected scale. Interactions calculated using percentage retention may lead to different interpretations and should not be treated as equivalent. A nonzero interaction contrast, supported by an appropriate statistical analysis and confidence interval, provides evidence that the combined effect differs from the sum of the individual effects. Such analyses are relevant to examining interactions between reprocessing and TiO_2_ incorporation, TiO_2_ and weathering, or FFF porosity and moisture exposure [29,33,77]. Figure 8 illustrates how molecular, structural, and component-level measurements can be combined to support interpretations of durability. A proposed degradation mechanism is more strongly supported when changes observed at different scales are consistent. Conversely, differences between molecular, structural, and mechanical results may reveal competing processes that require further investigation.

Table 7 summarizes the minimum characterization measurements recommended for evaluating PLA–TiO_2_ composites manufactured through FFF. It also identifies the principal questions addressed by each measurement and common interpretation errors that should be avoided.

## 9. Prospects for Future Research

Future research should move beyond separate evaluations of PLA recycling, TiO_2_ incorporation, printing parameters, and environmental exposure. Instead, studies should examine how material formulation, processing conditions, FFF architecture, and weathering jointly influence the properties and durability of printed components. The literature reviewed indicates that these factors can affect one another, but their interactions have rarely been investigated within the same material system. Addressing this limitation requires controlled experimental designs, measurements at different stages of processing and exposure, and independent validation under relevant environmental conditions.

Within the literature examined and the publication cutoff established for this review, no study was identified that systematically combines virgin and reprocessed PLA, controlled TiO_2_ characteristics, filament-fed FFF architecture, and time-resolved tropical weathering with comparable molecular, structural, and mechanical measurements.

Bergaliyeva et al. and Rodríguez et al. demonstrated the feasibility of manufacturing printed components containing reprocessed PLA and TiO_2_, but they did not investigate subsequent environmental weathering. Antunes et al. examined the accelerated weathering of PLA containing rutile TiO_2_, but their specimens were compression-moulded rather than produced through FFF, and the study did not evaluate previously reprocessed PLA. Other investigations, including those by Kakanuru and Pochiraju, Menezes et al., Klimczuk et al., and Faizaan et al., examined the degradation of printed PLA under different environmental conditions without simultaneously controlling TiO_2_ characteristics and previous reprocessing [29,30,32,46,90,91,109].

These studies provide important evidence for individual relationships among processing, material composition, printed architecture, and degradation. However, their differences in formulation, manufacturing methods, and exposure conditions prevent a quantitative assessment of the combined effects within a single material system. This limitation does not imply that no related investigations exist beyond the literature examined. Rather, it identifies the need for experiments capable of distinguishing individual effects from interactions among the variables of interest.

Table 8 summarizes these research priorities, the limitations of the existing evidence, and experimental approaches that could address them. The proposed order emphasizes the potential contribution of each priority to understanding causal relationships, improving comparisons between studies, and evaluating durability under tropical conditions. It should not be interpreted as a ranking based on the number of available publications.

The following experimental matrix provides a practical approach to addressing these research priorities by comparing controlled material formulations, FFF printing conditions, and environmental exposures.

The initial comparison should include virgin PLA and PLA subjected to a defined reprocessing procedure, both with and without TiO_2_. At least one low TiO_2_ concentration should be tested using particles with well-documented characteristics. If resources allow, a second concentration and a coated or phase-controlled TiO_2_ variant could provide further insight. To ensure reliable comparisons, all formulations should follow the same compounding and filament extrusion procedures, including a neat-PLA control subjected to identical processing conditions [29,30,32].

Beyond material composition, the printed structure should also be considered. An initial comparison could include two FFF architectures: one with low porosity and strong interlayer bonding, and another with a more connected pore network. Raster orientation and build direction should be selected to examine how interlayer bonding affects mechanical failure [12,13,45]. Both architectures should then be evaluated under different exposure conditions, including unexposed controls, temperature and humidity without UV, UV under dry conditions, combined UV–moisture cycles, and natural tropical weathering. Measurements at regular intervals would help identify changes in crystallinity, the onset of molar mass reduction, crack formation, and changes in failure behavior [44,46,102].

These experiments would also provide the data needed to understand how degradation develops over time. As discussed in Section 8, changes in molar mass distribution, crystallinity, oxidation, connected porosity, crack formation, and interlayer condition should be monitored throughout exposure. Rather than treating these measurements as separate indicators of ageing, their combined evolution can help explain how material degradation leads to a gradual loss of mechanical and functional performance.

The proposed framework can also support material selection and manufacturing decisions for PLA–TiO_2_ components intended for outdoor applications. Rather than relying solely on initial mechanical or thermal properties, different formulations and processing conditions should be compared based on their ability to retain the properties required for the intended application after environmental exposure. For reprocessed PLA, this approach can help identify processing strategies that limit molecular degradation while maintaining adequate printability and mechanical performance. For TiO_2_-containing formulations, it emphasizes the need to evaluate particle concentration, crystalline phase, surface treatment, and dispersion in relation to both initial properties and weathering behavior. Similarly, FFF manufacturing conditions can be assessed by considering their influence on interlayer bonding, void connectivity, moisture penetration, and mechanical failure. By relating these factors to changes in material structure and retained performance, the framework can help identify promising combinations of materials and processing conditions for outdoor applications. However, experimental validation is needed before specific design recommendations can be established.

This information can then support the development of models to predict the durability of FFF components. Such models should consider environmental conditions, changes in material properties, and the performance requirements of the intended application. Features such as pore connectivity, interlayer bonding, wall thickness, and weathering-induced cracks can affect moisture and oxygen transport. Therefore, models based only on the degradation behavior of bulk PLA may not accurately represent printed components. Similarly, TiO_2_ crystal phase, surface coating, and dispersion should be considered when assessing its influence on photochemical degradation.

Finally, these models should be developed using the time-dependent measurements and complementary characterization methods described in Section 8. Their predictions should then be validated against independent observations from natural tropical exposure. The results should identify the conditions under which components no longer meet their functional requirements and report uncertainties related to material batches, environmental conditions, and model parameters. Repeated experiments and data provided in reusable digital formats would further improve reproducibility and allow comparisons across different materials, printing conditions, and exposure environments.

## 10. Minimum Reporting Dataset

Improving the comparability and reproducibility of durability studies involving reprocessed PLA–TiO_2_ composites manufactured through FFF requires consistent reporting of material characteristics, processing conditions, printed architecture, and environmental exposure. Existing standards provide important guidance in specific areas. ISO/ASTM 52900 establishes terminology for additive manufacturing, while ISO 4892 and ASTM G151/G154/G155 describe principles and procedures for artificial weathering [8,39,41,42]. However, these standards do not individually address all the information needed to trace changes from recycled feedstock through filament production, printing, and environmental exposure. Table 9 therefore proposes a minimum reporting framework intended to support comparisons between studies rather than establish a new certification standard.

The proposed framework emphasizes measured values rather than nominal settings whenever these differences may affect the interpretation of experimental results. For example, a nominal infill of 100% does not demonstrate that a printed component is free of voids. Similarly, the nozzle temperature selected in the printer software does not necessarily represent the actual temperature experienced by the deposited material, and the particle size reported by a TiO_2_ supplier does not establish its dispersion within the printed composite [10,13,29]. Consistent reporting of these characteristics would help distinguish the effects of material formulation, processing, printed architecture, and environmental exposure, thereby improving reproducibility and the reliability of comparisons across studies.

## 11. Limitations

### 11.1. Limits of Existing Evidence

The available literature presents considerable variation in PLA grades, commercial additives, previous reprocessing, specimen geometry, build orientation, TiO_2_ crystalline phase and surface characteristics, exposure conditions, specimen conditioning, sample size, and reported properties [6,13,36,58]. These differences limit direct comparisons between studies, particularly when evaluating the magnitude of changes attributed to recycling, particle incorporation, or environmental exposure. In many cases, several variables change simultaneously, making it difficult to isolate their individual contributions or determine whether the reported differences result from material formulation, processing history, or exposure conditions. Accordingly, this review interprets the findings in relation to their underlying mechanisms, experimental conditions, and applicability to printed PLA-based components.

Many studies rely on relatively small sample sizes or a single material batch and report average properties without adequately examining variability associated with filament production, material batches, or different printers [29,46,54]. In addition, some investigations use commercial PLA+ formulations or colored filaments containing proprietary additives that may influence crystallization, UV absorption, hydrolysis, and fracture behavior [46,105]. Results obtained with these materials should therefore not be assumed to represent unmodified PLA.

Accelerated-weathering studies frequently employ UV-B, UV-C, or exposure cycles that are not fully documented. Although these conditions can provide useful comparative results and help identify degradation mechanisms, they cannot be directly translated into durability predictions for tropical environments without validation against natural outdoor exposure [28,34,39,43,46]. Conversely, natural-weathering studies are specific to their exposure locations and may lack complete measurements of spectral irradiance, radiant exposure, or time of wetness [45]. Both approaches provide valuable evidence, but neither independently establishes a universally applicable relationship between exposure conditions and component lifetime.

Incomplete documentation of weathering conditions introduces additional uncertainty. Lamp output, filter condition, irradiance calibration, interruptions during testing, specimen repositioning, and deviations from the planned exposure cycle can influence the actual conditions experienced by the specimens [41,42]. Without adequate records, differences in measured properties cannot always be attributed to well-characterized and reproducible exposure conditions. This limits quantitative comparisons even when the direction of the observed changes remains informative.

The interpretation of TiO_2_-related effects is particularly sensitive to differences in particle characteristics and processing conditions. Crystalline phase, surface coating, aggregation, processing-induced changes in PLA molar mass, pigmentation, and specimen thickness can all influence the response to environmental exposure [23,24,28,32]. Studies involving thin films or compression-moulded sheets provide useful information about chemical and physical degradation mechanisms, but their findings cannot directly establish the durability of FFF components. Similarly, results obtained from moulded specimens or pellet-fed printing require additional evaluation before being applied to filament-fed FFF components with different interlayer structures and exposure conditions.

### 11.2. Limits of This Review

The selection of sources was based on the scope of the review, the authors’ existing knowledge of the literature, searches in open academic databases, and access to published studies. Although the review includes relevant research, some publications may have been overlooked. Therefore, the findings should not be considered representative of the entire field. In addition, the focus on processing history, TiO_2_ characteristics, and FFF architecture may give less attention to other factors and approaches that influence PLA durability.

To improve the reliability of the analysis, the authors reviewed and discussed the selected studies, comparing their findings and interpretations. However, this process does not fully eliminate selection bias or independently confirm every numerical result reported in the literature. In some cases, only abstracts were available, limiting the verification of experimental details and conclusions. For this reason, agreement between studies is used to support reasonable interpretations rather than establish definitive conclusions. The proposed mechanisms and research priorities should therefore be examined further through additional literature and experimental validation.

The review also has clearly defined boundaries. It focuses on PLA durability under UV radiation, temperature, and moisture exposure, rather than providing a complete assessment of its environmental sustainability. Topics such as microbial biodegradation, toxicology, life-cycle assessment, chemical recycling, and other applications of TiO_2_ are not examined in detail. Although these topics are important, they fall outside the main objective of understanding how environmental exposure affects PLA performance during service. Biodegradation is discussed only when necessary to distinguish material degradation during use from its behavior at the end of its useful life [7,58,62].

## 12. Conclusions

This review examined the durability of PLA-based materials by critically analyzing the effects of reprocessing, TiO_2_ incorporation, FFF manufacturing, and environmental weathering. The findings show that the performance of printed components depends on the combined influence of material composition, processing conditions, printed architecture, and environmental exposure. These factors affect the initial characteristics of the material and its subsequent degradation, making durability difficult to assess through isolated measurements or individual processing variables.

The reviewed studies indicate that PLA reprocessing can alter molar mass, rheological behavior, crystallinity, and mechanical properties, although the magnitude of these changes depends on processing conditions and material characteristics. TiO_2_ incorporation can modify stiffness, thermal behavior, crystallization, and the response to UV radiation, but its effects depend strongly on crystalline phase, surface treatment, concentration, and dispersion. Consequently, improvements in initial mechanical or thermal properties do not necessarily indicate greater resistance to environmental degradation.

FFF manufacturing introduces additional factors that influence durability, particularly interlayer bonding, anisotropy, void connectivity, and component geometry. These structural characteristics can affect moisture penetration, crack development, and mechanical failure during weathering. The reviewed evidence also shows that degradation does not always produce a uniform reduction in material properties. Changes in crystallinity and internal structure may temporarily improve certain mechanical responses even as molecular degradation progresses. For this reason, reliable durability assessment requires the combined interpretation of molecular, chemical, thermal, structural, and mechanical measurements.

The main contribution of this review is the integration of these findings into a multiscale framework that connects processing history, TiO_2_ characteristics, FFF architecture, and environmental degradation with material-state changes and durability outcomes. While previous reviews have examined PLA recycling, PLA–TiO_2_ composites, additive manufacturing, and environmental degradation, these topics have generally been addressed separately or without fully considering their combined implications for the durability of printed components. This framework provides a basis for interpreting how these factors may interact across molecular, material, and component scales. In particular, it highlights how processing-induced changes in PLA properties can influence printed architecture, how TiO_2_ characteristics and structural features may modify environmental exposure and degradation, and how the resulting changes in material structure can affect mechanical performance and failure behavior. This integrated interpretation helps explain why changes in individual material properties do not necessarily reflect the overall durability of printed components. The review also proposes an experimental framework and minimum reporting requirements to support more consistent evaluation of PLA–TiO_2_ materials.

This work provides a foundation for investigating the long-term performance of reprocessed PLA–TiO_2_ components manufactured through FFF. The proposed approach emphasizes the need to evaluate processing history, material characteristics, printed architecture, and environmental exposure together, particularly under tropical conditions. By relating these factors to changes in material properties and performance after weathering, the framework can guide the comparison of material formulations, reprocessing strategies, and FFF manufacturing conditions. This perspective can support the development of more durable outdoor components by identifying material and processing combinations that retain adequate performance under the intended environmental conditions.

## Figures and Tables

**Figure 1:**
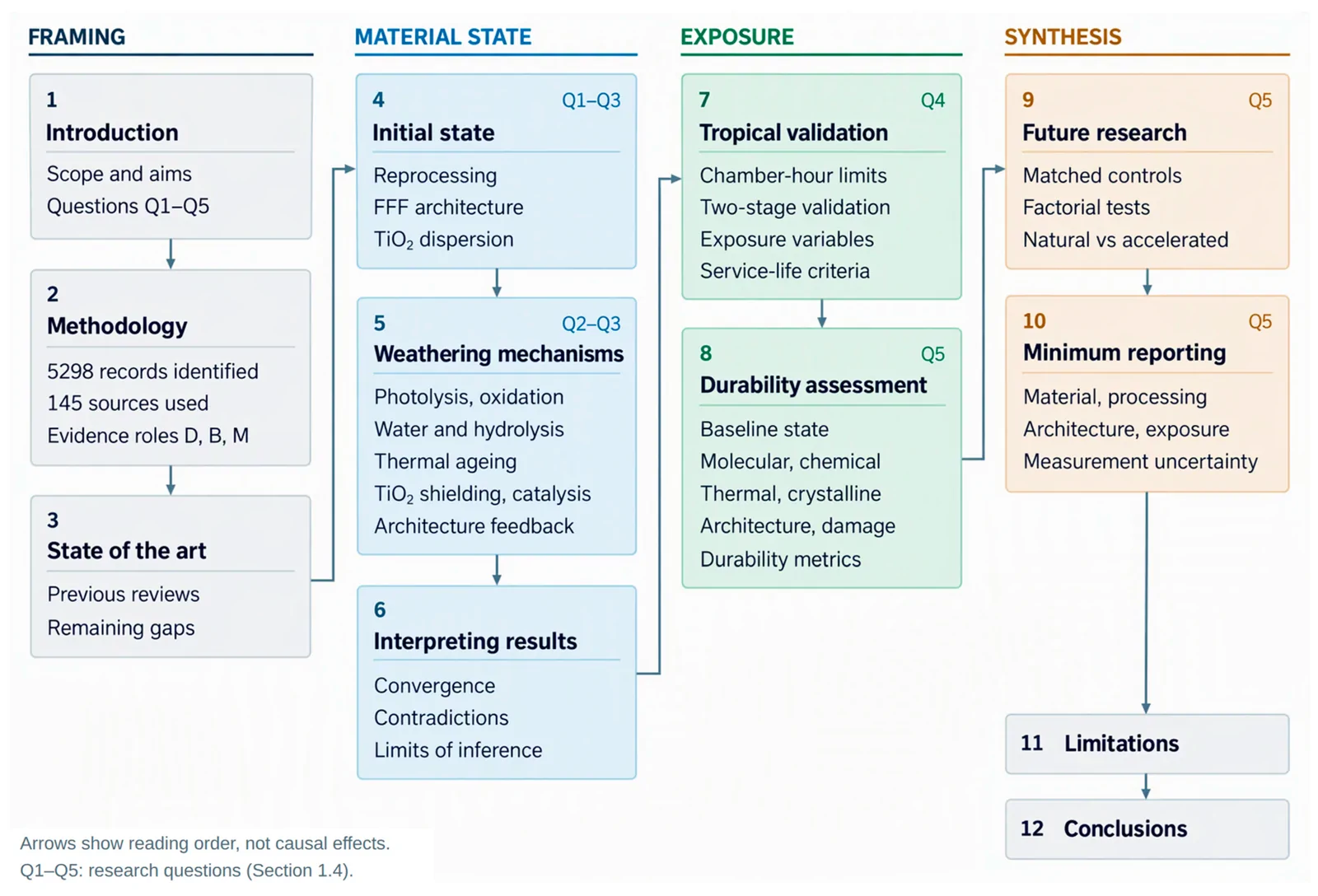
Thematic roadmap of the review. Sections are grouped as framing (Section 1, Section 2 and Section 3), material state and mechanisms (Section 4, Section 5 and Section 6), exposure and assessment (Section 7 and Section 8), and synthesis (Section 9, Section 10, Section 11 and Section 12). Arrows show reading order, not established causal effects; Q1–Q5 refer to the research questions in Section 1.4.

**Figure 2:**
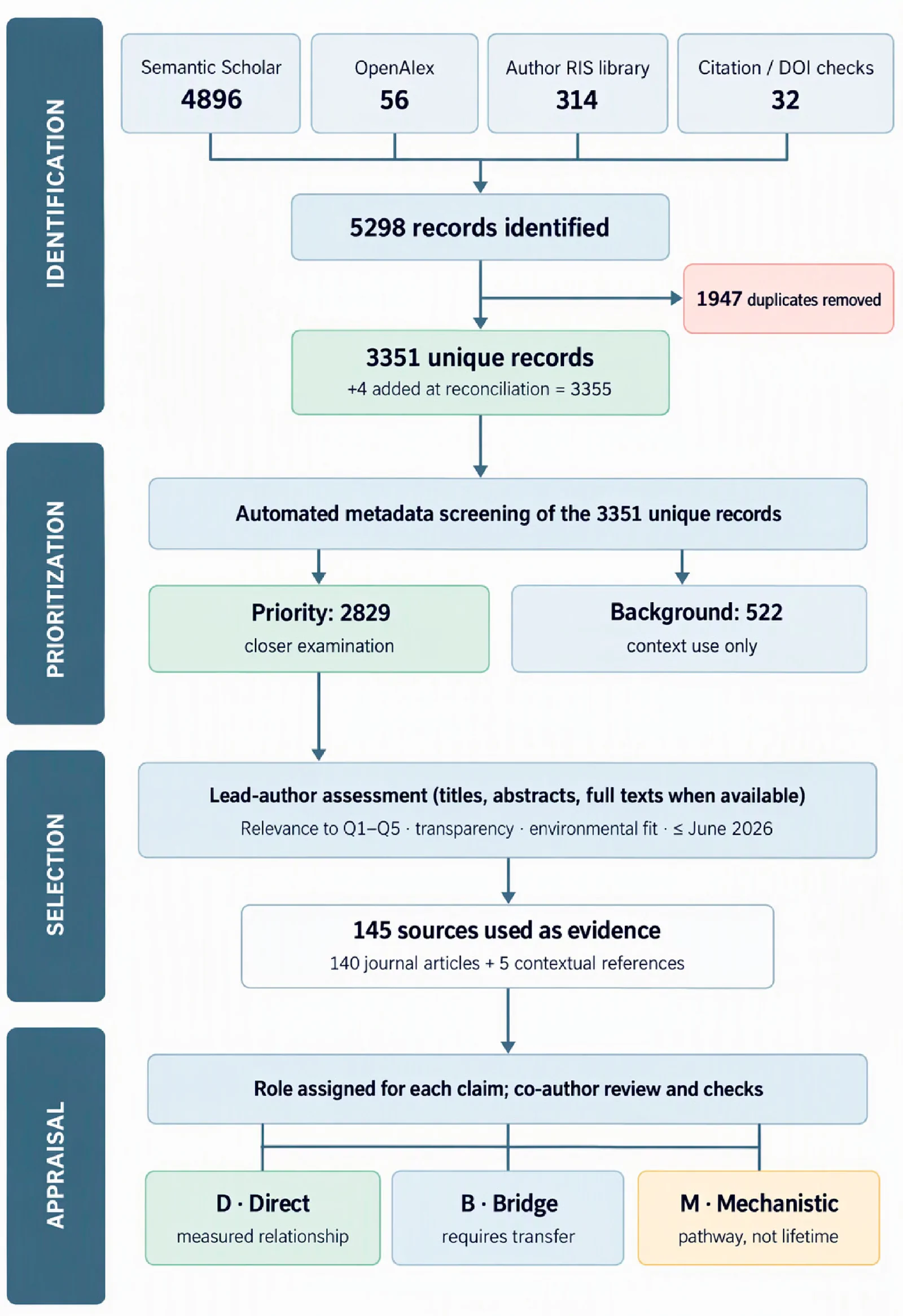
Flow of literature identification, prioritization, selection, and evidence appraisal. Prioritization labels come from automated metadata screening and are not inclusion or exclusion decisions.

**Figure 3:**
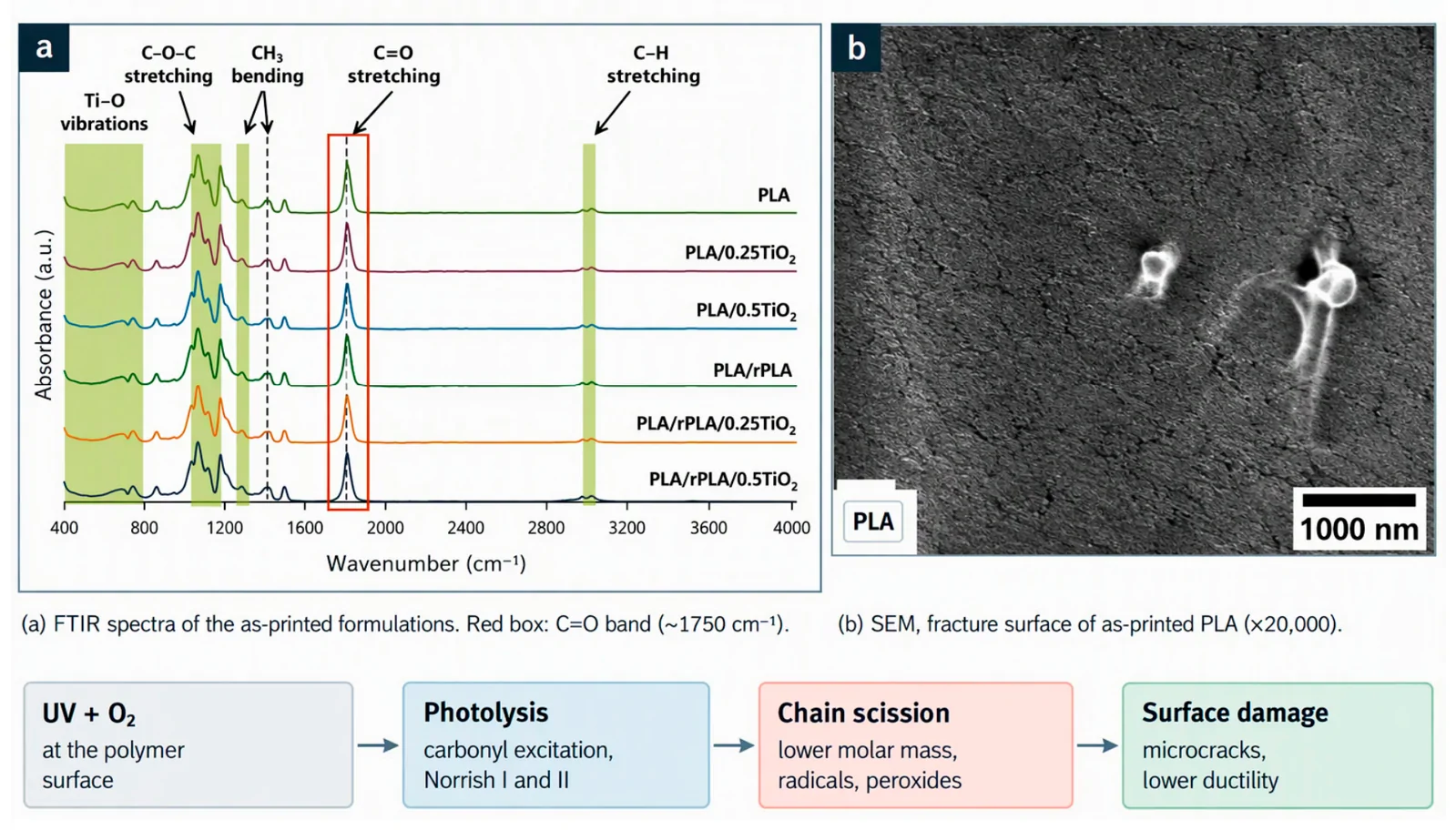
Photolysis and photo-oxidation of PLA. (**a**) FTIR spectra, adapted with permission from Ref. [30]. 2026, and (**b**) SEM fracture surface of as-printed specimens (authors’ unpublished data, 2026) as pre-exposure reference; the sequence below summarizes the mechanism in Section 5.1 [24,34,35,36,38].

**Figure 4:**
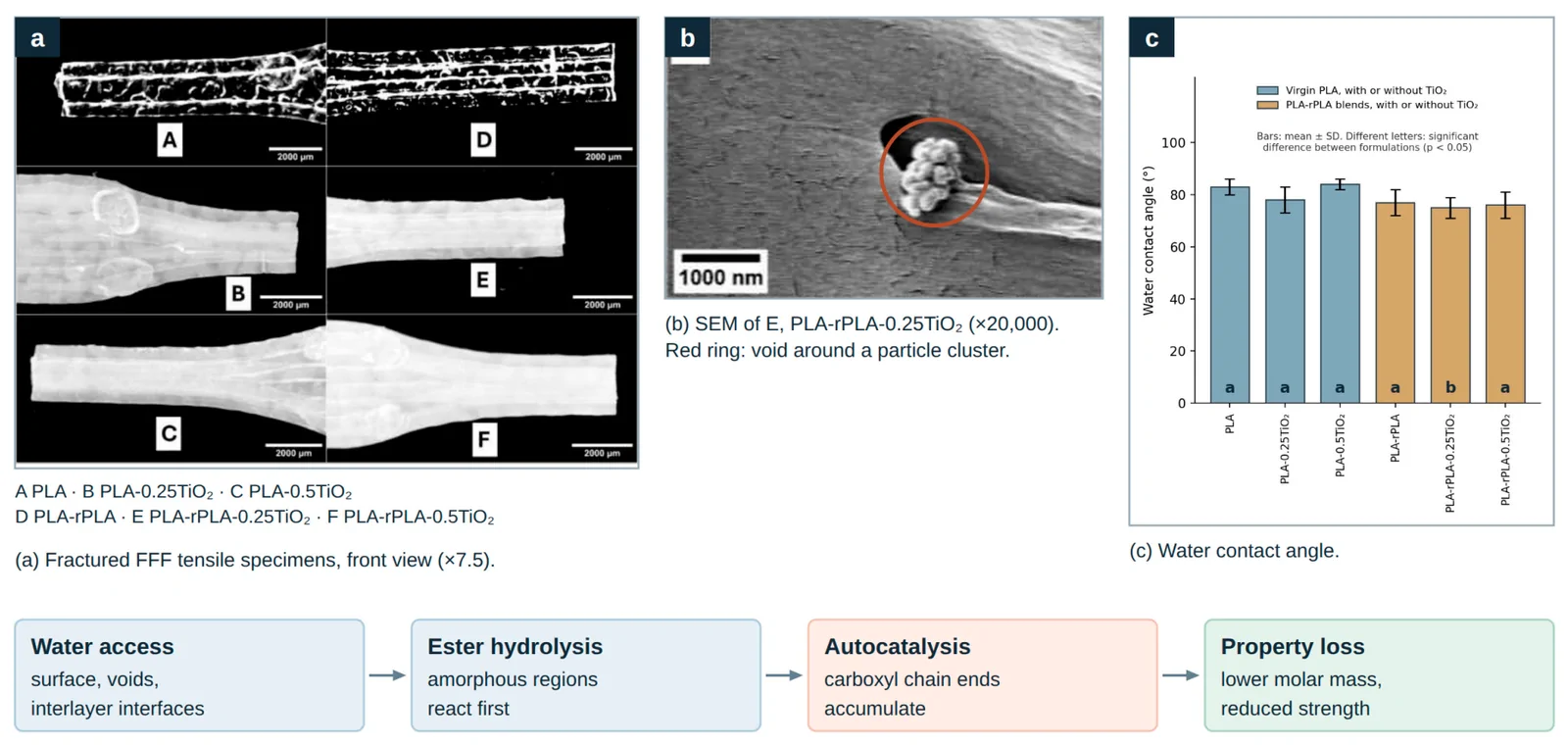
Water transport and autocatalytic hydrolysis in PLA. (**a**) Fractured FFF specimens, (**b**) SEM of a void around a particle cluster (authors’ unpublished data, 2026) and (**c**) water contact angle of as-printed specimens, where blue bars show virgin PLA formulations, tan bars show PLA-rPLA blends, error bars show standard deviation, and different letters indicate significant differences (*p* < 0.05), adapted with permission from Ref. [30], 2026; the sequence below summarizes the mechanism in Section 5.2 [5,102,103,119,120,121,122].

**Figure 5:**
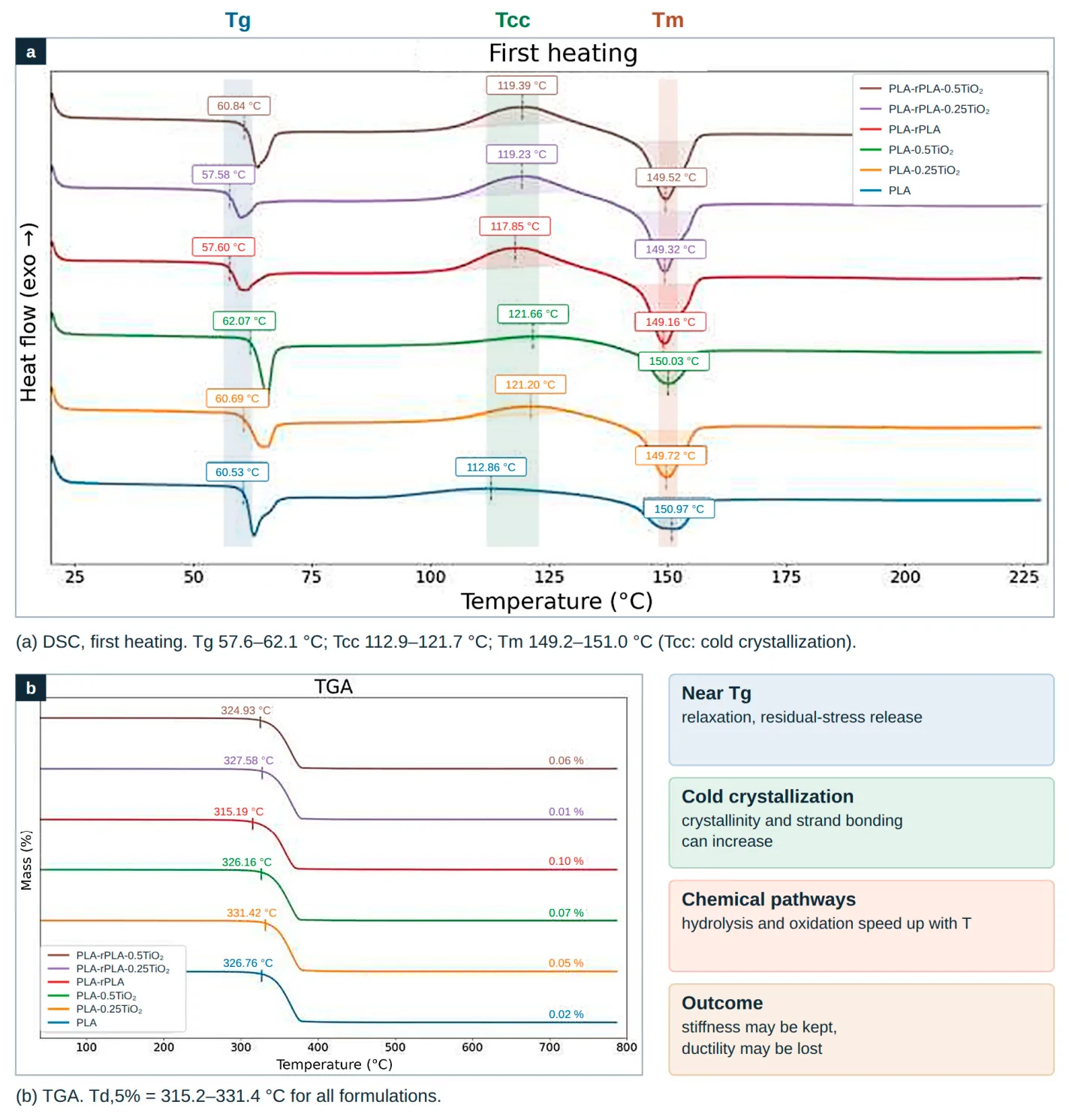
Thermal ageing and annealing of PLA. (**a**) DSC first heating, where the upward arrow on the heat-flow axis indicates the exothermic direction, and (**b**) TGA of as-printed specimens (authors’ unpublished data, 2026); the sequence below summarizes the consequences dis-cussed in Section 5.3 [46,111,126,127,128].

**Figure 6:**
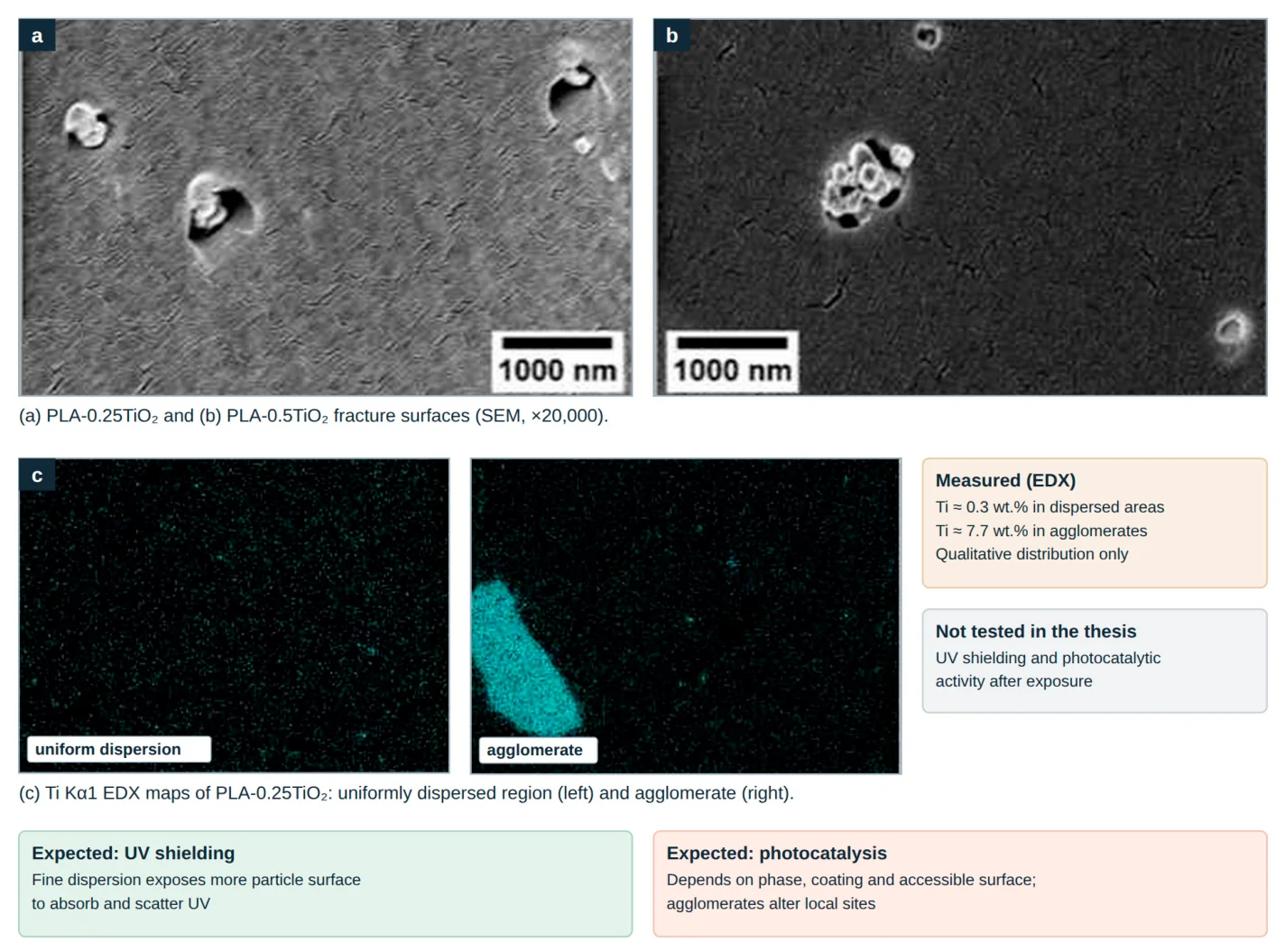
TiO_2_ in PLA: dispersion, UV shielding, and photocatalysis. (**a**,**b**) SEM and (**c**) EDX Ti map of as-printed PLA–TiO_2_ specimens (authors’ unpublished data, 2026); UV shielding and photocatalytic activity after exposure were not measured. Related evidence is in [24,32,93,133,134,135].

**Figure 7:**
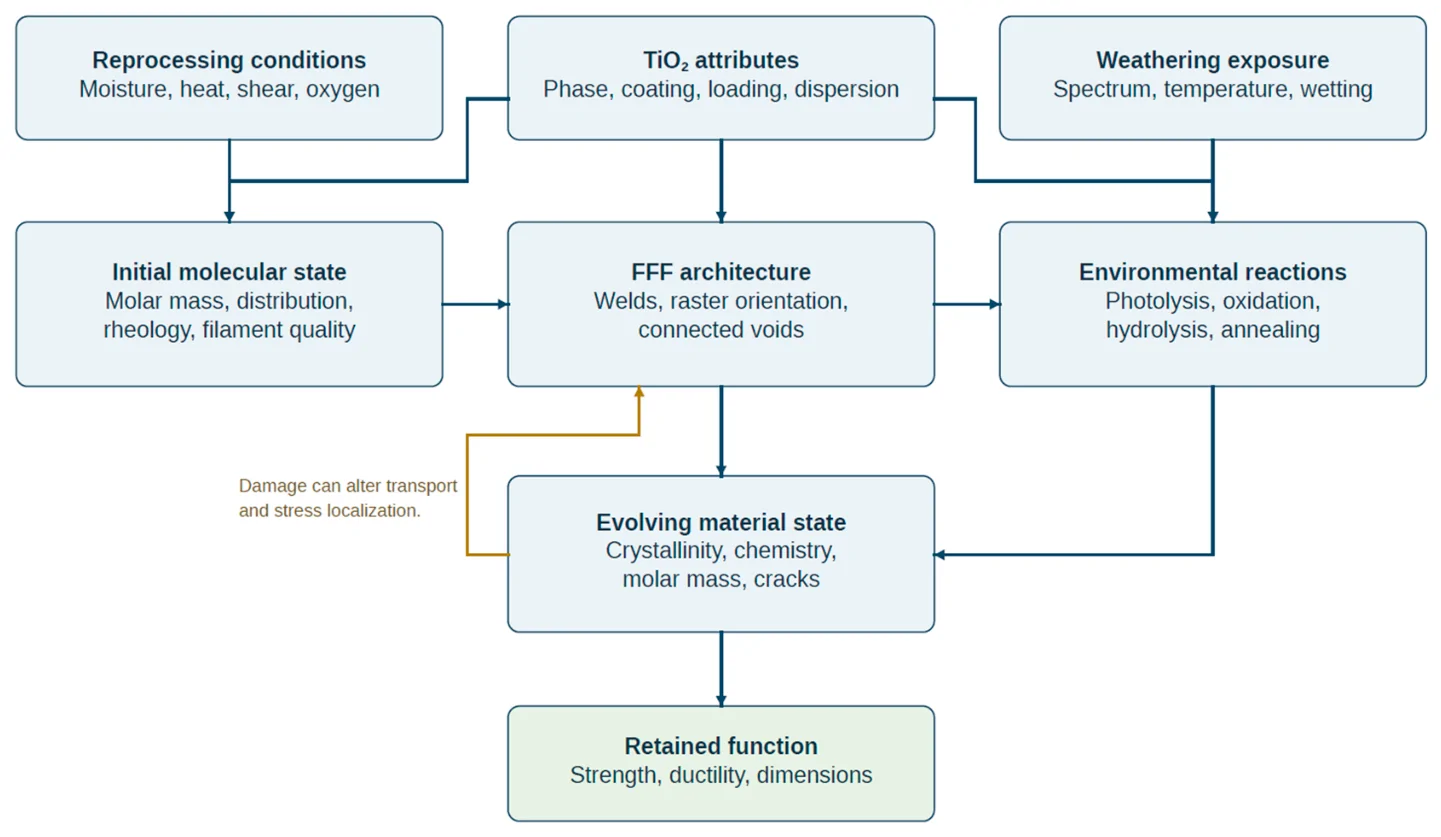
Proposed multiscale causal chain. Blue-gray blocks represent input factors, material and architectural states, and processes; the green block represents the retained function (outcome). Dark blue arrows represent testable dependencies rather than universal effect directions, and the gold arrow indicates a feedback in which damage can alter transport and stress localization [6,9,12,13,32]. Abbreviations: TiO_2_, titanium dioxide; FFF, fused filament fabrication.

**Figure 8:**
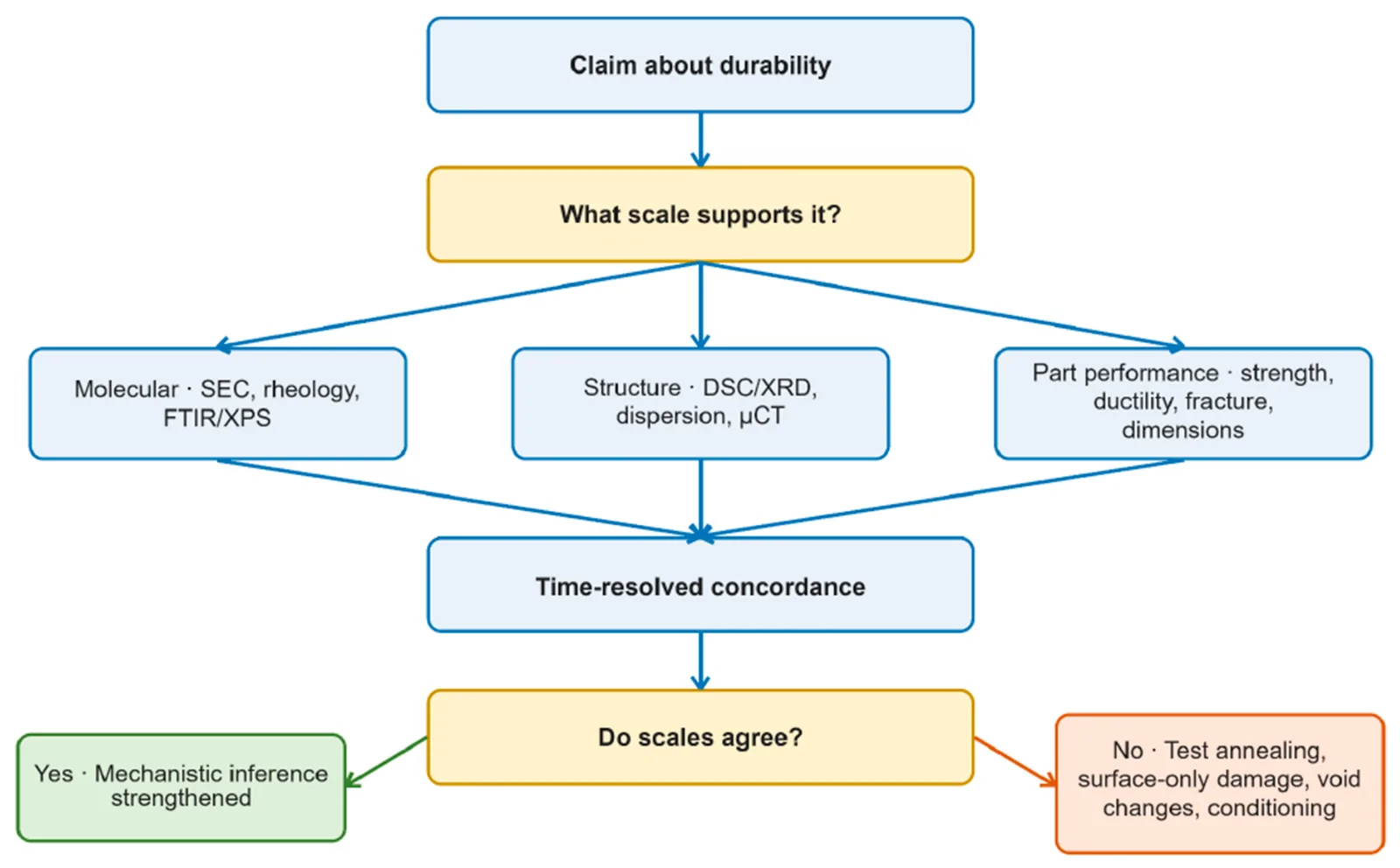
Integration of molecular, structural, and component-level evidence for evaluating durability. Blue blocks represent the claim, the measurement scales, and the time-resolved comparison; yellow blocks represent decision questions; the green block and arrow indicate that the scales agree, and the orange block and arrow indicate that they do not, with the follow-up checks listed. Consistent changes across measurement scales strengthen mechanistic interpretations, while differences between measurements may help identify competing degradation processes [29,32,46,111]. Abbreviations: SEC, size-exclusion chromatography; FTIR, Fourier-transform infrared spectroscopy; XPS, X-ray photoelectron spectroscopy; DSC, differential scanning calorimetry; XRD, X-ray diffraction; µCT, micro-computed tomography.

**Table 1:** Representative thematic domains and search terms used across bibliographic platforms.

Search Domain	Representative Terms
PLA reprocessing	PLA; recycling; reprocessing; multiple extrusion; molar mass; rheology
Material extrusion	FFF; material extrusion; filament; interlayer; weld; porosity
TiO_2_ modification	titanium dioxide; rutile; anatase; coating; dispersion
Environmental durability	weathering; UV; hydrolysis; humidity; outdoor; tropical
Combined domains	reprocessed PLA; FFF; TiO_2_; irradiation; durability
Previous reviews	PLA; recycling; additive manufacturing; TiO_2_; weathering; review

**Table 2:** Comparative assessment of reviewed studies.

Review	Main Scope and Contribution	Limitation Relative to the Present Review	Evidence Gap Retained
Pérez-Fonseca et al. (2023) [59]	PLA recycling, reprocessing, and prioritization; PLA/TiO_2_ preparation, compatibility, mechanical, thermal, photocatalytic, and antimicrobial properties.	Scope assessed from publisher abstract only. Predominantly non-FFF systems; limited service-weathering synthesis; no FFP/weathering synthesis.	No integrated TiO_2_–FFF–weathering analysis. Phase, coating, dispersion, and print architecture are not integrated with durability.
Solek et al. (2026) [60]	AI polymer processing degradation and durability standards. Accelerated weathering methods and photodegradation of PLA and biocomposites.	Abstract-level comparison; material-specific coverage unverified. Limited treatment of FFF-specific interfaces and recycled feedstock; evidence predates recent MEX studies.	Combined rPLA/TiO_2_–FFF topical weathering remains unresolved. No architecture-aware tropical service model.
Kaseem et al. (2019) [25]	PLA/TiO_2_ preparation and multifunctional properties. Mechanisms, characterization, and enhancement of interlayer bonding in FFF polymers.	Predominantly non-FFF systems; limited recycling and weathering analysis. Focuses on bond recycling. Does not address environmental exposure; not PLA/TiO_2_ or recycling-specific.	TiO_2_ attributes and FFF architecture are not linked to durability. No link between weld quality, environmental ingress, and weathered retention.
Sola and Trinchi (2023) [61]	Recycling of composite feedstocks and FFF parts. Formation, imaging, and quantification of voids in FFF parts.	Emphasizes recyclability rather than outdoor performance. Does not synthesize weathering chemistry or processed PLA/TiO_2_.	Prior reprocessing is not linked to weathered-property retention. Void connectivity is recognized as a transport path but rarely coupled to ageing.
Hasan et al. (2024) [58]	Mechanical and sustainability aspects of recycled PLA printing. Influence of process parameters on mechanical properties of FFF PLA.	Limited TiO_2_-specific and long-term weathering analysis. Mechanical-property emphasis; heterogeneous feedstocks; little post-weathering evidence.	Outdoor durability of printed rPLA nanocomposites remains unresolved. Process optimization is not equivalent to durability optimization.
González-López et al. (2020) [36]	Accelerated weathering and photodegradation of PLA biocomposites. Broad review of PLA feedstock, FDM parameters, properties, and applications.	Limited coverage of rPLA and FFF interfaces. Weathering, recycling history, and TiO_2_ photochemistry are not jointly analyzed.	No architecture-aware tropical durability framework. PLA printability evidence remains separated from service durability.
Singh et al. (2025) [62]	Recycled and reinforced thermoplastics for sustainable FDM. Framework for mutually dependent material requirements in FFF feedstocks.	Broad polymer scope; limited weathering and TiO_2_ photochemistry. General materials-design focus rather than PLA ageing.	Reinforcement benefits lack long-term exposure assessment. Durability and recyclate history are not normalized as coupled printability constraints.
Velghe et al. (2023) [6]	PLA melt degradation, characterization, and stabilization. Overview of thermoplastic-filament rheology and FDM process requirements.	Excludes service weathering and most FFF architecture. Multi-polymer and predominantly pre-service scope.	Processing damage is not traced from printed-part exposure. Rheological printability metrics are not followed through environmental ageing.
Sandanamsamy et al. (2023) [56]	PLA feedstocks, FDM parameters, properties, and applications. Mechanisms, variables, measurement, and stabilization of PLA during melt processing.	Recycling, TiO_2_, and weathering are not jointly assessed. Service weathering, biodegradation, and most additive-manufacturing architecture are out of scope.	Printability remains disconnected from service durability. Processing damage is not followed into printed-part exposure.
Cojocaru et al. (2022) [63]	Effects of FFF parameters on PLA mechanical properties. Mechanical and sustainability potential of recycled PLA in 3D printing.	Primarily pre-weathering mechanical analysis. Limited TiO_2_-specific and long-term weathering analysis; sustainability evidence uneven across dimensions.	Process optimization is not linked to durability retention. Recyclability is not connected to outdoor durability or nanoparticle-mediated ageing.
Frigione and Rodríguez-Prieto (2021) [64]	Accelerated ageing and long-term prediction of polymers. Weathering of plastics and biocomposites, including mechanical changes and VOC emissions.	Broad polymer scope; no target formulation or architecture. Broad polymer scope; limited FFF and PLA/TiO_2_ resolution.	Mechanism equivalence and field transfer require validation. Emissions and fragmentation are rarely quantified for printed rPLA nanocomposites.
Nzimande et al. (2024) [65]	Weathering of plastics and biocomposites, including mechanical changes and VOC emissions. PLA biodegradation mechanisms, microorganisms, analytical methods, and test standards.	Limited FFF and PLA/TiO_2_-specific evidence. End-of-life biodegradation rather than service durability; FFF architecture is peripheral.	Fragment and emission release from printed rPLA nanocomposites remains unresolved. Durability-degradability trade-offs remain unconnected to functional retention.
Tao et al. (2021) [13]	Formation, imaging, and quantification of FFF voids. Recycled and reinforced thermoplastics for sustainable FDM.	Excludes PLA/TiO_2_ weathering chemistry. Multi-polymer scope; weathering and TiO_2_ photochemistry are not central.	Void connectivity is rarely coupled to environmental ageing. Reinforcement benefits are not evaluated against long-term environmental exposure.
Gao et al. (2021) [12]	FFF interlayer bonding mechanisms and characterization. PLA recycling, reprocessing, and prior weathering, as stated in its abstract.	Focuses on pre-exposure bonding; not PLA/TiO_2_-specific. Scope comparison based on the publisher abstract; no exhaustive full-text comparison claimed.	Weld quality is not linked to environmental ingress and durability. Need for a controlled TiO_2_–FFF–weathering synthesis.
Acierno and Patti (2023) [57]	Filament rheology and FDM processing requirements. Recycling of polymer-composite feedstock and printed parts in FFF.	Multi-polymer scope, mainly before service exposure. Circularity and technical feasibility do not establish outdoor life.	Rheological changes are not tracked through weathering. Link between recyclate history and retained function under exposure remains unresolved.
Sola (2022) [16]	Interdependent material requirements for FFF feedstocks. Accelerated-ageing procedures and long-term prediction across polymer environments.	General materials-design framework; not PLA ageing. Broad polymer scope rather than the target formulation and architecture.	Effects of prior processing on durability remain unresolved. Validation of mechanism equivalence and field transfer is needed.
Lors et al. (2025) [7]	PLA biodegradation mechanisms, methods, and standards. AM polymer degradation, processing effects, and durability standards, as stated in its abstract.	Addresses end-of-life degradation rather than service performance. Abstract-level scope comparison; not a verified assessment of all target-system evidence in its full text.	Durability-degradability trade-offs lack functional assessment. Resolves the specific rPLA/TiO_2_–FFF–tropical intersection only partially.

**Table 3:** Experimental scope and limits of transfer in six representative studies [29,30,32,45,46,54]. D, direct evidence for the specific relationship discussed; B, bridge evidence connecting relevant domains with a transfer limitation; M, mechanistic analogy informing mechanisms without quantitative transfer. Evidence role indicates applicability to the present synthesis rather than methodological quality. Abbreviations: PLA, polylactic acid; rPLA, reprocessed PLA; PLA+, commercial modified PLA; FFF, fused filament fabrication; TiO_2_, titanium dioxide.

Study	Material/Fabrication	Exposure	Key Evidence	Evidence Role/Transfer Limit
Anderson (2017) [54]	PLA/recycled PLA; Filament-fed FFF	Before weathering	Mean properties largely retained, but recycling increased variability.	D–Direct reprocessing–FFF evidence; one recycling route and no weathering.
Bergaliyeva et al. (2023) [29]	PLA/rPLA + TiO_2_; Direct pellet printing	Before weathering	TiO_2_ response depended on virgin/reprocessed formulation.	B–Links rPLA, TiO_2_, and printing; pellet-fed route and no weathering.
Rodríguez et al. (2026) [30]	PLA/rPLA + TiO_2_; Filament-fed FFF	Before weathering	Low-loading composite filaments are printable; thermomechanical gains reported.	D–Direct evidence for the target initial state; weathering not tested.
Antunes et al. (2020) [32]	PLA + rutile TiO_2_; Compression-moulded sheets	Accelerated UVA + condensation	TiO_2_ system developed earlier crystallinity and severe surface damage.	B–Direct PLA/TiO_2_ weathering evidence, but non-FFF geometry and unmatched initial molar mass.
Faizaan et al. (2026) [46]	Commercial PLA + Filament-fed FFF	Accelerated UVB + humidity	Time-dependent structural changes and tensile-strength loss.	D–Direct FFF weathering evidence; proprietary PLA and aggressive UV-B limit transfer.
Chopra et al. (2023) [45]	PLA with varied infill; Filament-fed FFF	Natural atmospheric exposure	Printed architecture influenced degradation and tensile response.	D–Direct natural-weathering evidence; one formulation and exposure site.

**Table 4:** Primary studies providing direct, bridge, or mechanistic evidence across the review domains. D, direct evidence for the specific relationship discussed; B, bridge evidence connecting relevant domains with a transfer limitation; M, mechanistic analogy used to inform degradation or transport mechanisms without quantitative transfer to the target system. Evidence role indicates applicability to the present synthesis rather than methodological quality.

Study	Material and Process	Exposure or Main Comparison	Key Evidence	Evidence Role/Transfer Limit
Bergaliyeva et al. (2023) [29]	PLA/75 wt.% rPLA; 0–7 wt.% TiO_2_; pellet printing	Reprocessing + TiO_2_	TiO_2_ improved horizontal strength in PLA blends but reduced it in virgin PLA; crystallinity decreased.	B–Strong rPLA–TiO_2_ link; pellet-fed and unweathered.
Rodríguez et al. (2026) [30]	PLA/rPLA; 0.5 wt.% TiO_2_; filament FFF	Reprocessing + low TiO_2_	Printable filaments; selected stiffness and thermal gains with TiO_2_.	D–Target material/process initial state; no weathering.
Yılmaz (2026) [31]	PLA/TiO_2_; FFF at 180–240 °C	TiO_2_ + print temperature	TiO_2_ increased crystallinity and flowability; temperature partly offset mechanical losses.	B–Relevant TiO_2_–FFF interaction; no rPLA or weathering.
Antunes et al. (2020) [32]	PLA/3 wt.% rutile-TiO_2_; moulded sheets	UVA-340 + condensation, 2000 h	TiO_2_ system showed earlier crystallization, cavities, yellowing, and sorbed-water damage.	B–Direct TiO_2_ weathering evidence; non-FFF and unweathered initial molar mass.
Alfaro et al. (2022) [44]	FFF PLA scaffolds	Heated cyclic temperature–UVA–humidity	Combined stressors produced the strongest spectroscopic and morphological changes.	D–Direct coupled-ageing evidence in FFF; scaffold-specific geometry.
Chopra et al. (2023) [45]	FFF PLA; varied infill	Natural outdoor exposure	Infill affected degradation and tensile response.	D–Direct natural FFF weathering; site- and formulation-specific.
Faizaan et al. (2026) [46]	Commercial PLA + FFF coupons	UV-B + condensation, 2000 h	Chain-scission signatures and crystallinity increased while strength declined.	D–Time-resolved FFF weathering; proprietary PLA and aggressive UV-B.
Lee et al. (2022) [49]	Recycled PLA filament	Recycling	Thermal and mechanical response changed with recycling.	B–Relevant recycled-feedstock evidence; no weathering or TiO_2_.
Anderson (2017) [54]	PLA; print–grind–re-extrude–reprint	Virgin vs. recycled	Recycling caused modest mean changes but greater variability and clogging.	D–Direct reprocessing FFF evidence; no TiO_2_ or weathering.
Agbakoba et al. (2024) [71]	Virgin/waste PLA blends; FFF	Waste source + fraction	Some failed-print blends retained stability; high waste fraction reduced strength.	D–Direct recycled-PLA FFF evidence; mixed waste chemistries confound cycle effects.
Hidalgo-Carvajal et al. (2023) [72]	PLA wastes repeatedly extruded to films/filaments	Waste origin + recycled-filament extrusion	Feedstock provenance altered recycled-filament behaviour.	B–Relevant processing evidence; no finished-part weathering.
Janutėnienė et al. (2024) [77]	Virgin/recycled PLA, ABS, PETG; material extrusion	Recycling and separate UV exposure	Recycled variants were generally more brittle.	B–Connects recycling, printing, and UV, but not in one factorial PLA design.
Jirků et al. (2025) [78]	PLA/PLA–coffee grounds; print–UV–recycle–reprint	UV + recycling + filler	Pre-irradiation and filler identity affected reprocessed-part performance.	B–Strong sequence evidence; filler is not a TiO_2_ analogue.
Kakanuru and Pochiraju (2020) [90]	FFF PLA, ABS, PLA/SiC	Water at 50/70 °C	Moisture, filler loading, and architecture affected retained tensile response.	B–Printed-part transport evidence; SiC and hot immersion differ from target weathering.
Klimczuk et al. (2024) [91]	3D-printed PLA structures	Acid/alkaline hydrolysis at 90 °C	Linked architecture, molecular change, crystallinity, and erosion.	M–Strong mechanistic evidence under severe non-service conditions.
Algarni (2021) [92]	FDM PLA; varied raster	Moisture conditioning	Raster and moisture jointly affected mechanical response.	B–Architecture-moisture link; conditioning rather than ageing.
Bergaliyeva et al. (2022) [102]	FFF PLA	Thermal/hydrothermal ageing	Hydrothermal ageing reduced tensile performance more than hot ageing.	D–Direct FFF ageing evidence; one PLA grade and laboratory-bound condition.
Ekinci et al. (2021) [103]	Single-layer FFF PLA films	Hydrolysis at 37 °C, 280 d	Molar mass, modulus, and strength fell while crystallinity increased.	B–Links molecular and mechanical evolution; biomedical temperature and monolayer geometry.
Alfaro et al. (2022) [104]	FFF PLA scaffold	ASTM F1980/G154	Functional torque failure used to estimate shelf life.	B–Useful functional-endpoint example; device-specific acceleration and failure criterion.
Amza et al. (2021) [105]	FFF PLA/PETG	Dry UV-B, 24 h	PLA tensile and compressive strengths decreased; stiffness changed little.	D–Direct printed-PLA UV response; very short dry exposure.
Souissi et al. (2023) [106]	UV-aged PLA filament → FFF	Pre-ageing + raster + water uptake	Pre-printing degradation carried into printed mechanical/moisture response.	B–Useful carryover evidence; filament, not finished part, was UV-aged.
Upadhyay et al. (2020) [107]	FFF PLA specimens/propeller	Simulated marine exposure, 30 d	Mechanical, wear, toughness, and pitting responses changed with exposure.	D–Direct printed-part marine response; short immersion and no natural validation.
Nieto et al. (2021) [108]	FFF PLA/PETG	Water and saturated solutions, 10 wk	Printed discontinuities increased absorption relative to bulk expectations.	D–Direct architecture-transport evidence; solutions are not rainfall/seawater analogues.
Menezes et al. (2022) [109]	Additively manufactured PLA	Artificial seawater, 22–60 °C	Part-scale diffusion, mechanics, and Arrhenius analysis were linked.	D–Direct printed-part hydrothermal evidence; no field validation or mechanism-equivalence test.
Puerta et al. (2023) [110]	FDM PLA/biodegradable ABS analogue	Simulated sunlight or saltwater, 8 wk	Saltwater progressively reduced PLA maximum stress; light response was non-monotonic.	D–Direct printed-PLA exposure evidence; non-standard solar simulation was interrupted by lamp failures.
Tejedor et al. (2025) [111]	Annealed/non-annealed FFF PLA	Saltwater, 45 °C, 45 d	Annealing reduced voids and water uptake and mitigated property loss.	D–Direct architecture-water durability evidence; no UV or field validation.
Sabalina et al. (2025) [112]	FFF PBS/PLA and PBS/PHB	Artificial seawater, 6 mo	Orientation and defects affected sorption, hydrolysis, crystallinity, and mechanical loss.	B–Architecture evidence in PLA-containing blends; not neat PLA/TiO_2_.
Castañón-Jano et al. (2025) [113]	Printed PLA/metal-filled PLA	UV/rain vs. pH 13 at 50 °C	Alkaline exposure caused greater damage; defects accelerated degradation.	B–Printed PLA ageing evidence; application and high filler contents differ strongly from the target system.

**Table 5:** Interpretation matrix for recurring apparent contradictions.

Observation A		Observation B	Reconciliation Variables That Must Be Tested	Required Measurements
Crystallinity rises after reprocessing or weathering [22,46].	Crystallinity falls with increasing TiO_2_ in some printed composites [29].	Chain length, cooling rate, phase and coating of TiO_2_, dispersion, thermal protocol, normalization.		DSC first/second heat, XRD/WAXS, molar mass, cooling profile.
Recycled PLA retains near-virgin average strength [54].	High recycled fraction causes substantial strength loss in another formulation [71].	Waste provenance, contamination, stabilizers, drying, cycle severity, filament variability, voids.		SEC, rheology, moisture, filament diameter, μCT, strength distribution.
Combined UV-humidity weathering lowers strength over time [46].	Deliberate annealing can reduce voids and mitigate selected salt-water losses [111].	Exposure sequence, spectrum and dose, temperature relative to glass transition, duration, formulation, raster.		Time-resolved strength and elongation, DSC/XRD, density/μCT, failure mode, SEC.
TiO_2_ improves thermal or mechanical metrics before exposure [29,30].	TiO_2_ accelerates surface damage during UVA/moisture weathering [32].	Photocatalytic accessibility, phase, surface coating, initial molar mass, oxygen/water, aggregation.		Phase analysis, XPS/TEM/SEM mapping, radical or oxidation metrics, SEC, retained properties.
Higher printing temperature improves welding [10,12].	Higher thermal exposure can accelerate PLA degradation [6,20].	Time-temperature-shear integral, moisture, oxygen, residence time, molecular relaxation.		In-process temperature, residence time, rheology/SEC, interlayer fracture, void fraction.
Annealing-derived crystallinity lowers water uptake [111].	Weathering-derived crystallinity accompanies chain scission and embrittlement [46,138].	Crystal form, lamellar organization, molar mass, void change, post-treatment, exposure sequence.		DSC plus WAXS/XRD, SEC, μCT, sorption, toughness, dimensional stability.
Clay improves selected retained properties in an accelerated cycle [126].	Clay accelerates photo-oxidation during natural exposure [139].	Filler chemistry, dispersion, spectrum, wetting and humidity conditions, outdoor site, property versus chemical endpoint.		Natural-accelerated paired exposure, SEC/FTIR, dispersion mapping, retained strength and toughness.
A controlled weld approaches bulk strength [85,86].	Printed parts remain anisotropic and environmentally sensitive [87,90].	Net section, grooves, raster, void connectivity, weld quality, ageing-induced fracture relocation.		Interlayer-specific fracture, DIC, μCT, actual road geometry, failure-location mapping.

**Table 6:** Weathering approaches and their appropriate evidentiary role.

Approach	Strength	Principal Limitation	Minimum Information for Comparison	Appropriate Claim
Natural outdoor exposure [43,45]	Highest environmental realism for the site and orientation.	Slow; variable; not automatically transferable across locations or years.	Coordinates, dates, orientation, irradiance/radiant exposure, temperature, RH, rainfall/time of wetness, specimen surface temperature, pollutants.	Site-specific durability and chamber correlation.
Fluorescent UVA plus condensation [32,40,41]	Reproducible control of short-wave UV, temperature, and wetting.	Narrow spectrum; condensation is not equivalent to all tropical wetting; chamber hours are not service time.	Lamp type and age, irradiance and control wavelength, cycle, black-panel temperature, RH/condensation, spray water, repositioning.	Comparative accelerated weathering under the reported cycle.
UV-B plus humidity [46,105]	Strong acceleration and time-resolved mechanistic sensitivity.	Contains a more aggressive short-wavelength distribution than terrestrial sunlight.	Spectral peak/distribution, irradiance, dose, cycle temperature, humidity, exposure side, specimen flipping.	Mechanistic ranking; not unvalidated tropical lifetime.
Filtered xenon arc [42,142]	Broader spectral approximation to sunlight; configurable moisture.	Filter, irradiance, temperature, and water cycles can produce substantially different results.	Lamp/filter combination, spectral irradiance, radiant exposure, black-panel temperature, chamber/air temperature, RH, spray cycle.	Comparative solar-simulation evidence when fully specified.
Thermal/humidity ageing without light [33,44]	Isolates hydrolysis, physical ageing, and annealing.	Cannot reproduce photochemical pathways or UV-moisture synergy.	Temperature, RH or water activity, duration, conditioning, oxygen, specimen geometry.	Mechanistic component of a factorial design.

**Table 7:** Recommended multiscale characterization package.

Scale	Minimum Measurement	Question Answered	Common Misinterpretation
Feedstock	Grade, prior use, D-content if available, additives, measured moisture [2,6]	What material entered each melt step?	Treating commercial colour or trade name as a complete formulation.
Molecular	SEC/GPC and oscillatory rheology at defined stages [6,20]	Did chains shorten and did processability change?	Inferring molar mass solely from tensile strength or melt-flow rate.
Chemical surface	ATR-FTIR with a stated index and acquisition protocol; XPS where needed [35,46]	Which surface functional groups evolve?	Treating surface spectra as bulk composition.
Thermal/crystalline	DSC first and second heat; XRD/WAXS [29,32]	Did manufacturing or exposure alter order and transitions?	Calling higher crystallinity unconditionally beneficial.
TiO_2_	Phase, size, coating, loading, and statistically representative dispersion mapping [25,26]	Which particle system is present and where?	Assuming equal behaviour for all TiO_2_ powders.
FFF architecture	Density plus μCT or validated image analysis; bead/layer dimensions [13,15]	What void topology and weld geometry control transport and failure?	Using slicer infill as measured porosity.
Surface/damage	Colour and gloss, profilometry, SEM/AFM, crack density [32,44]	Where does damage initiate and propagate?	Generalizing from one selected micrograph.
Performance	Strength, modulus, elongation, toughness, failure location; creep/fatigue where relevant [12,46]	Does the part retain its functional response?	Equating strength increase with increased durability.
Exposure	Spectrum, irradiance, radiant exposure, surface temperature, RH/wetting, orientation [39,41]	What environmental dose was applied?	Comparing chamber hours across unlike protocols.

**Table 8:** Research questions and proposed experimental priorities.

Priority	Gap	Evidence Limitations	Recommended Design
1	Reprocessing × TiO_2_ × tropical weathering interaction	The discussed studies provide partial evidence, but do not establish the full interaction [29,30,32].	Full or response-surface factorial design with identically processed controls and repeated exposure intervals.
2	TiO_2_ phase and surface coating	Rutile, anatase, particle size, functionalization, and co-stabilizers produce different optical and chemical responses [98,131,133,134].	Compare characterized rutile, anatase, and coated controls at matched size/loading/dispersion.
3	Dispersion topology versus total loading	Processing route and agglomerate localization can dominate local stress, crystallization, and photochemistry [26,93,96].	Quantitative multi-scale mapping linked to local oxidation and crack initiation.
4	Connected FFF porosity as a transport network	Slicer infill and 2D micrographs cannot quantify 3D ingress pathways [13,45,90,91].	μCT before/after exposure, water uptake, diffusion modelling, and interlayer fracture.
5	Time-resolved non-monotonic response	Terminal tests miss competing crystallization, void evolution, and embrittlement [46,111].	Dense early intervals plus longer exposure; change-point or nonlinear mixed-effects models.
6	Natural-accelerated correlation in a tropical site	Field results are site-specific and chamber hours have no universal conversion [39,43,139,140].	Co-expose reference materials and identical printed formulations outdoors and in calibrated chambers.
7	Filament production and variability	Recycled filament diameter, moisture, and contamination affect print defects [54,72].	Inline diameter logging, moisture measurement, rheology, and lot-level random effects.
8	Functional failure criteria	A 50% retention threshold, aqueous Arrhenius prediction, or scaffold shelf life is not universal [104,109,143].	Define application-specific limit states for strength, deflection, dimensions, and fracture mode.
9	Molecular-to-part lifetime modelling	Existing molecular and aqueous kinetic models omit changing FFF transport and TiO_2_ photochemistry [52,144,145].	Couple molecular scission, sorption, crystallization, damage, and failure; validate against independent field data.
10	Fatigue, creep, and fracture	Most weathering studies emphasize monotonic strength [12,13,107].	Weathered interlayer fracture, fatigue, creep, and wear under relevant temperature/humidity.
11	Fragment and additive release	Weathered plastics can emit volatile products and fragments, but printed PLA/TiO_2_ emissions are rarely quantified [36,65].	Collect particles, oligomers, VOCs, and titanium during weathering; distinguish release from accessibility.

**Table 9:** Minimum information to report.

Domain	Required Fields
PLA identity	Manufacturer and grade; batch; D-lactide or optical purity if known; declared additives/pigments; initial Mn, Mw, dispersity, and moisture.
Recycling history	Waste origin; contamination control; washing and drying; number and type of melt-processing cycles; extruder geometry; set and measured melt temperatures; screw speed; throughput; residence time or distribution; atmosphere.
TiO_2_	Supplier; crystalline phase and method of verification; primary and aggregate size; specific surface area if available; surface coating/treatment; purity; loading; dispersion procedure and quantitative dispersion evidence.
Filament	Extrusion conditions; cooling and take-up; diameter mean, variation, and sampling frequency; ovality; moisture before printing; storage and conditioning.
FFF	Printer and firmware; nozzle material/diameter; actual and set temperatures; chamber/bed conditions; speed; flow multiplier; layer height/width; raster, infill, orientation, perimeters; specimen location; post-treatment.
Initial architecture	Part density; total and connected void fraction; bead/layer dimensions; surface roughness; representative microscopy or μCT; failure-critical interface orientation.
Weathering	Standard and exact cycle; lamp/filter and spectrum; irradiance and control wavelength; radiant exposure; black-panel/specimen temperature; air temperature; RH; condensation/spray/rain and water quality; specimen orientation, flipping, and repositioning.
Natural exposure	Coordinates; elevation; dates; rack angle/azimuth; global and UV irradiance; rainfall; RH; air and surface temperature; time of wetness; pollution or salt context; controls.
Conditioning/testing	Pre- and post-exposure conditioning; time between exposure and measurement; specimen number; randomization; test standard; loading rate; full stress–strain or relevant functional response; failure location.
Statistics and data	Replicate definition; power or sample-size rationale; model and interaction terms; effect size and confidence interval; missing data; raw or machine-readable data; image-analysis workflow.

## Data Availability

No new data were created or analyzed in this study. Data sharing is not applicable to this article.

## Abbreviations

ABS	Acrylonitrile–butadiene–styrene
AFM	Atomic force microscopy
AM	Additive manufacturing
ASTM	ASTM International
ATR-FTIR	Attenuated total reflectance Fourier-transform infrared spectroscopy
DIC	Digital image correlation
DOI	Digital object identifier
DSC	Differential scanning calorimetry
EDX	Energy-dispersive X-ray spectroscopy
FDM	Fused deposition modeling
FFF	Fused filament fabrication
FTIR	Fourier-transform infrared spectroscopy
GPC	Gel permeation chromatography
ISO	International Organization for Standardization
MEX	Material extrusion
PBAT	Poly(butylene adipate-co-terephthalate)
PBS	Poly(butylene succinate)
PETG	Glycol-modified poly(ethylene terephthalate)
PHB	Polyhydroxybutyrate
PLA	Poly(lactic acid)
PLA+	Commercial modified PLA
rPLA	Reprocessed PLA
PLLA	Poly(L-lactic acid)
RH	Relative humidity
SEC	Size-exclusion chromatography
SEM	Scanning electron microscopy
Tcc	Cold crystallization temperature
Td	Temperature at 5% mass loss
TEM	Transmission electron microscopy
TGA	Thermogravimetric analysis
Tg	Glass transition temperature
TiO_2_	Titanium dioxide
UV	Ultraviolet
UV-A (UVA)	Ultraviolet A
UVA-340	UVA-340 fluorescent lamp
UV-B	Ultraviolet B
UV-C	Ultraviolet C
VOC(s)	Volatile organic compound(s)
WAXD	Wide-angle X-ray diffraction
WAXS	Wide-angle X-ray scattering
XPS	X-ray photoelectron spectroscopy
XRD	X-ray diffraction
μCT	Micro-computed tomography
